# The activation of OsEIL1 on *YUC8* transcription and auxin biosynthesis is required for ethylene-inhibited root elongation in rice early seedling development

**DOI:** 10.1371/journal.pgen.1006955

**Published:** 2017-08-22

**Authors:** Hua Qin, Zhijin Zhang, Juan Wang, Xinbing Chen, Pengcheng Wei, Rongfeng Huang

**Affiliations:** 1 Biotechnology Research Institute, Chinese Academy of Agricultural Sciences, Beijing, China; 2 National Key Facility of Crop Gene Resources and Genetic Improvement, Beijing, China; 3 Rice Research Institute, Anhui Academy of Agricultural Sciences, Hefei, China; National University of Singapore and Temasek Life Sciences Laboratory, SINGAPORE

## Abstract

Rice is an important monocotyledonous crop worldwide; it differs from the dicotyledonous plant *Arabidopsis* in many aspects. In *Arabidopsis*, ethylene and auxin act synergistically to regulate root growth and development. However, their interaction in rice is still unclear. Here, we report that the transcriptional activation of OsEIL1 on the expression of *YUC8/REIN7* and indole-3-pyruvic acid (IPA)-dependent auxin biosynthesis is required for ethylene-inhibited root elongation. Using an inhibitor of YUC activity, which regulates auxin biosynthesis *via* the conversion of IPA to indole-3-acetic acid (IAA), we showed that ethylene-inhibited primary root elongation is dependent on YUC-based auxin biosynthesis. By screening phenotypes of seedling primary root from mutagenesis libraries following ethylene treatment, we identified a rice ethylene-insensitive mutant, *rein7-1*, in which YUC8/REIN7 is truncated at its C-terminus. Mutation in *YUC8/REIN7* reduced auxin biosynthesis in rice, while *YUC8/REIN7* overexpression enhanced ethylene sensitivity in the roots. Moreover, YUC8/REIN7 catalyzed the conversion of IPA to IAA, truncated version at C-terminal end of the YUC8/REIN7 resulted in significant reduction of enzymatic activity, indicating that YUC8/REIN7 is required for IPA-dependent auxin biosynthesis and ethylene-inhibited root elongation in rice early seedlings. Further investigations indicated that ethylene induced *YUC8/REIN7* expression and promoted auxin accumulation in roots. Addition of low concentrations of IAA rescued the ethylene response in the *rein7-1*, strongly demonstrating that ethylene-inhibited root elongation depends on IPA-dependent auxin biosynthesis. Genetic studies revealed that YUC8/REIN7-mediated auxin biosynthesis functioned downstream of OsEIL1, which directly activated the expression of *YUC8/REIN7*. Thus, our findings reveal a model of interaction between ethylene and auxin in rice seedling primary root elongation, enhancing our understanding of ethylene signaling in rice.

## Author summary

Rice is an important crop worldwide and is grown in water-saturated environments during its life cycle. This unique feature confers that rice might have different aspects from *Arabidopsis* in ethylene signaling. Although the crosstalk between ethylene and auxin is well understood in *Arabidopsis*, however, the interaction in rice is largely unclear. Here, we show that YUC8/REIN7, a member of the *YUC* gene family, catalyzing the conversion of IPA to IAA in auxin biosynthesis, is transcriptionally modulated by ethylene signaling component OsEIL1, and mainly participates in auxin biosynthesis and ethylene-inhibited root growth. We first identified that ethylene-inhibited root elongation is suppressed by the inhibitor of YUC activity, and YUC8/REIN7 is required for IPA-dependent auxin biosynthesis, indicating that YUC8/REIN7 is involved in ethylene-inhibited root elongation in rice early seedlings. Moreover, ethylene induced *YUC8/REIN7* transcription and promoted auxin accumulation in roots. Addition of low concentrations of IAA rescued the ethylene response in the *rein7-1*, demonstrating that ethylene stimulates auxin biosynthesis dependent on YUC8/REIN7 function. Further evidence revealed that OsEIL1 transcriptionally activates the expression of *YUC8/REIN7*, and YUC8/REIN7-mediated auxin biosynthesis genetically acts downstream of OsEIL1. Our data in the present report identified an interaction between ethylene and auxin in rice seedling primary root elongation, increasing our understanding of ethylene signaling in rice root growth.

## Introduction

Root systems of higher plants play essential roles in absorbing water and nutrients and supporting the plant body. Improved root architecture is crucial for productivity and a very important contributor to extract water under water-limited stress [1]. The dicotyledonous plant *Arabidopsis* has a primary root and lateral roots, whereas monocotyledonous crops, including rice (*Oryza sativa* L.), have fibrous root systems composed of a primary root, lateral roots and adventitious roots. The primary root, initiated during embryo development, develops shortly after germination and is a fundamental part of the root system that absorbs mineral nutrients, and provides mechanical support for shoot growth [2–4]. Root development is affected by diverse endogenous and exogenous factors, such as ethylene and auxin, which are central regulators of this process [5–9].

Previous studies have shown that auxin biosynthesis, transport and auxin-dependent signaling affect root development [10–12]. Indole-3-acetic acid (IAA), the major form of auxin in plants, can be biosynthesized in tryptophan (Trp) -dependent and -independent pathways [13,14]. There are four pathways for IAA biosynthesis from Trp in plants: the YUCCA (YUC) pathway or the indole-3-pyruvic acid (IPA) pathway, the tryptamine (TAM) pathway, the indole-3-acetamide pathway, and the indole-3-acetaldoxime pathway [13]. The YUC pathway has been proposed as the most important pathway to produce auxin in plant [15–17], and the YUC family of flavin-containing monooxygenases and the TRYPTOPHAN AMINOTRANSFERASE OF ARABIDOPSIS (TAA) family of aminotransferases are key enzymes in this pathway [13,18–20]. YUC catalyzes the conversion of IPA to IAA, a rate-limiting step in the IPA pathway [15,16]. The diversity of auxin biosynthesis indicates that different pathways may have distinctive roles in plant tissue growth and development.

Ethylene is a simple and very important gaseous phytohormone that modulates multiple plant growth and developmental processes [21]. In *Arabidopsis*, ethylene signaling is started from the perception by a family of endoplasmic reticulum-located receptors [22,23]. In the absence of ethylene, the active receptors recruit a Raf-like protein kinase, CONSTITUTIVE TRIPLE RESPONSE 1 (CTR1), to phosphorylate the C-terminal domain of ETHYLENE INSENSITIVE 2 (EIN2), which represses the downstream ethylene response [24–27]. In the presence of ethylene, ethylene binding to the receptors inhibits the interaction with CTR1, resulting in that CTR1 cannot phosphorylate EIN2. Unphosphorylated EIN2 is cleaved by an unknown kinase, and the EIN2 C-terminus translocates into the nucleus [26,28,29], to stabilize the transcription factors EIN3 and EIN3-LIKE1 (EIL1), which are sufficient and necessary for activation of many ethylene-response genes. These changes ultimately cause different physiological responses [30,31].

Ethylene is known to inhibit root elongation [5,7]. This regulation is revealed to mediate the interaction with auxin [7,19,32–35]. For example, auxin increases 1-aminocyclopropane-1-carboxylic acid synthase (ACS) gene transcription and ethylene biosynthesis [36]. Similarly, ethylene application promotes the expression of IAA biosynthetic genes and IAA levels [19,33,34]. Furthermore, mutants related to auxin biosynthesis, distribution, or signaling display abnormal responses to ethylene [34,37–39], and the genes involved in local auxin biosynthesis, such as *ANTHRANILATE SYNTHASE α1* (*ASA1*), *ANTHRANILATE SYNTHASE β1* (*ASB1*), *TAA1* and *TRYPTOPHAN AMINOTRANSFERASE-RELATED PROTEIN 1* (*TAR1*), which are regulated by ethylene, exhibit ethylene-insensitive root growth [19,34]. YUC genes also play an important role in root responses to ethylene [16]. Recent studies have shown that several factors, such as EIN3, ETHYLENE RESPONSE FACTOR 1 (ERF1) and PHYTOCHROME INTERACTING FACTOR 4 (PIF4), function as crosstalk nodes between ethylene and auxin in primary root elongation [9,40,41], suggesting that ethylene-inhibited primary root growth in *Arabidopsis* requires auxin biosynthesis, transport, or signaling.

Rice is one of the most common crops worldwide and grown in water-saturated environments during its life cycle. Ethylene plays important roles in rice adaption to hypoxic conditions [42]. In the dark, ethylene promotes coleoptile growth of rice seedlings but inhibits root elongation [43,44]. The double response in rice is different from the triple response of dark-grown *Arabidopsis* seedlings, which have inhibited hypocotyls and roots, with an exaggerated apical hook [21]. Although rice has five receptors, including two members [ETHYLENE RESPONSE SENSOR 1 (OsERS1) and OsERS2] of subfamily I and three members [ETHYLENE RESPONSE 2 (OsETR2), OsETR3 and OsETR4] of subfamily II [45,46], an ETR1-type receptor is absent compared to *Arabidopsis* [44]. Moreover, a single loss-of-function mutation of ethylene receptors in rice can lead to phenotypic changes, whereas multiple receptor loss-of-function mutations in *Arabidopsis* can cause major phenotypic changes [23,47,48]. As discussed above, CTR1 is a key negative regulator of ethylene signaling [24]. *Arabidopsis* contains a single CTR1, whereas there are three CTR-like genes in rice, and ethylene receptor signal output is mediated in part by OsCTR2 [49]. Rice has six EIN3-like homologues, and only OsEIL1 and OsEIL2 are involved in the ethylene signaling [50]. OsEIL1 and OsEIL2 spatially regulate the ethylene response of roots and coleoptiles of etiolated seedlings, which differs from the incomplete ethylene insensitivity of *ein3* and *eil1* in *Arabidopsis* [50,51]. In rice, ethylene triggers root-specific accumulation of abscisic acid (ABA), which is required for root inhibition [48,52]. This ethylene-ABA interaction mode is different from previous reports, in which ABA negatively regulates ethylene production and then inhibits root growth in *Arabidopsis* [53–55]. These findings suggest that different features are present in rice ethylene signaling. Although the crosstalk between ethylene and auxin has been well studied in *Arabidopsis*, their interaction in rice is largely unclear. In this study, we report that YUC8/REIN7, a member of the YUC gene family, located genetically downstream of OsEIL1, is mainly involved in the conversion of IPA to IAA in auxin biosynthesis and root growth. Thus, the results provide a new insight into understanding of ethylene and auxin interaction in regulating rice root growth.

## Results

### YUC-based auxin biosynthesis is required for ethylene-inhibited root elongation

Ethylene promotes coleoptile growth of etiolated rice seedlings but inhibits root elongation [43,44,48,50,52], which is known as the double response. To determine whether the double response of ethylene in rice is conserved in different genetic backgrounds, we treated three *japonica* cultivars and three *indica* cultivars with ethylene. All genotypes exhibited increased coleoptile growth and inhibited root elongation (S1A–S1C Fig), suggesting that the ethylene double response is common in etiolated rice seedlings. Next, we investigated whether ethylene biosynthesis or signaling is involved in this process. We assessed the effect of the ethylene biosynthesis inhibitor 1-aminoethoxyvinyl-glycine (AVG) and ethylene competitive inhibitor 1-methylcyclopropene (1-MCP) on coleoptile and root growth in the presence of ethylene. Our data showed that the 1-MCP, but not AVG, suppressed the ethylene response of etiolated *japonica* and *indica* seedlings (S1A–S1C Fig). To further examine the requirements for the ethylene signaling pathway in this process, we assessed the ethylene double response in ethylene signaling mutants. Our results revealed that the *osein2* mutant was insensitive to ethylene in both root elongation and coleoptile promotion, while *oseil1* was insensitive to ethylene in root elongation, but showed normal coleoptile promotion S2A–S2C Fig). Correspondingly, overexpression of *OsEIN2*/*OsEIL1* (EIN2-OX and EIL1-OX, respectively) resulted in an enhanced ethylene response following ethylene treatment (S2A–S2C Fig). These data indicate that ethylene-promoted coleoptile growth and ethylene-inhibited root elongation are primarily mediated by the ethylene signaling pathway.

**Fig 1:**
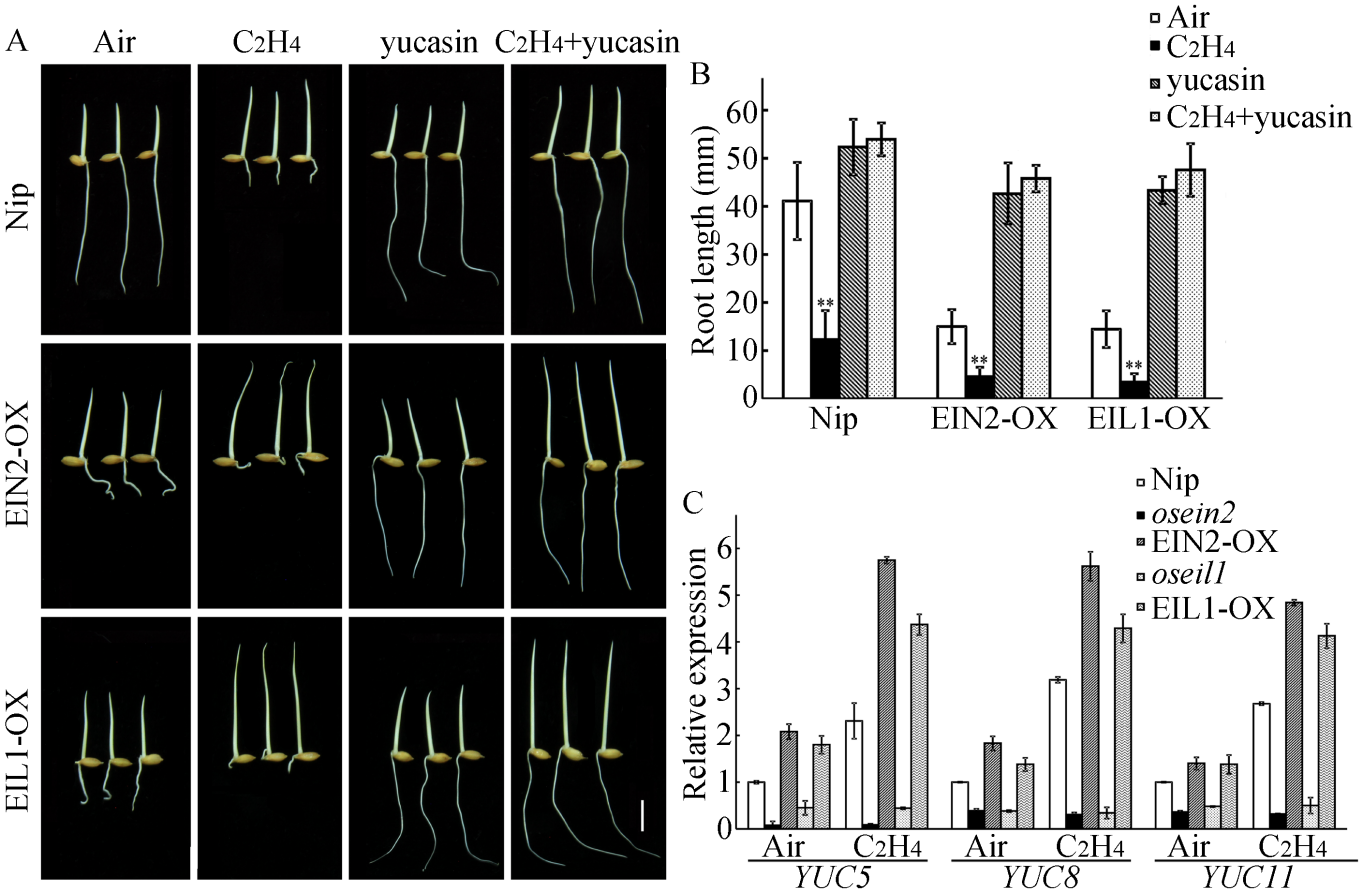
YUC-dependent auxin production is required for ethylene-inhibited root growth. (A) Primary root phenotypes of Nipponbare (Nip), overexpressing *OsEIN2* (EIN2-OX) and overexpressing *OsEIL1* (EIL1-OX) transgenic lines treated with or without 10 ppm ethylene, in the absence or presence of 10 μM yucasin. Rice seedlings were grown in the dark for 3 d. Bar = 10 mm. (B) Root length of the plants shown in (A). Values are shown as the mean ± SD of 20–30 seedlings. The experiment was repeated at least three times with similar results. ** indicates significant difference compared to air at *P* < 0.01. (C) Expression of YUC genes in 3-d-old etiolated seedling roots. 3-d-old etiolated seedlings were treated with or without 10 ppm ethylene for 3 h. The RNAs from roots were isolated for qPCR. The experiment was repeated at least five times with similar results. Bars indicate ± SD.

Multiple reports have demonstrated that ethylene-inhibited *Arabidopsis* root growth is mediated through the effects on auxin biosynthesis [9,19,33,34]. And YUC controls the rate-limiting step of auxin biosynthesis *via* the conversion of IPA to IAA [15,16]. There are 14 and 11 YUC genes in rice and *Arabidopsis*, respectively. To determine whether YUC-based auxin biosynthesis is required for ethylene-inhibited root growth in rice, we inhibited YUC activity with chemical inhibitor. Our observations showed that ethylene-inhibited root growth in the wild-type seedlings was suppressed by yucasin [5-(4-chlorophenyl)-4H-1,2,4-triazole-3-thiol], an inhibitor of YUC activity, in the presence of ethylene (Fig 1A and 1B). This suppression of ethylene-inhibited root elongation by yucasin was further confirmed using the overexpression transgenic lines EIN2-OX and EIL1-OX (Fig 1A and 1B). We then detected the expression of *YUC* genes in seedling roots. Our results showed that ethylene induced the expression of most *YUC* genes (Figs 1C and S3). Especially, the induction of *YUC5*, *YUC8*, and *YUC11* was dependent on OsEIN2 and OsEIL1 (Fig 1C). These differential inducible characters might be due to the temporal and spatial expression patterns of *YUC* genes, some of them may be not involved in ethylene-inhibited root elongation in early seedlings, thus some *YUC* genes are not regulated by OsEIN2 and OsEIL1 in roots. Alternatively, the expression of *YUC3* was obviously induced by ethylene, even in the absence of *osein2* (S3 Fig), implying that there might have an OsEIN2-independent ethylene response pathway in rice. These findings indicate that ethylene-inhibited root elongation is mainly dependent on YUC activity-based auxin biosynthesis, and *YUC5*, *YUC8*, and *YUC11* may be involved in ethylene-inhibited root elongation.

### YUC8, a flavin-containing monooxygenase, is required for IPA-dependent auxin biosynthesis

To identify the factors involved in ethylene-inhibited root elongation, we conducted a screen for ethylene-insensitive mutants from our mutagenesis (generated with fast neutron bombardment and chemical induction) and T-DNA insertion libraries in rice [56,57] in the dark. At least 7 rice ethylene-insensitive (*rein*) mutants were selected based on the phenotypes of the roots and coleoptile of etiolated seedlings (S4A Fig). Compared to the wild type, all the mutants, such as *rein7-1*, showed insensitivity or reduced sensitivity to ethylene in root elongation but exhibited normal coleoptile growth (S4B and S4C Fig), indicating that these mutation genes may be involved in ethylene-inhibited root elongation.

**Fig 2:**
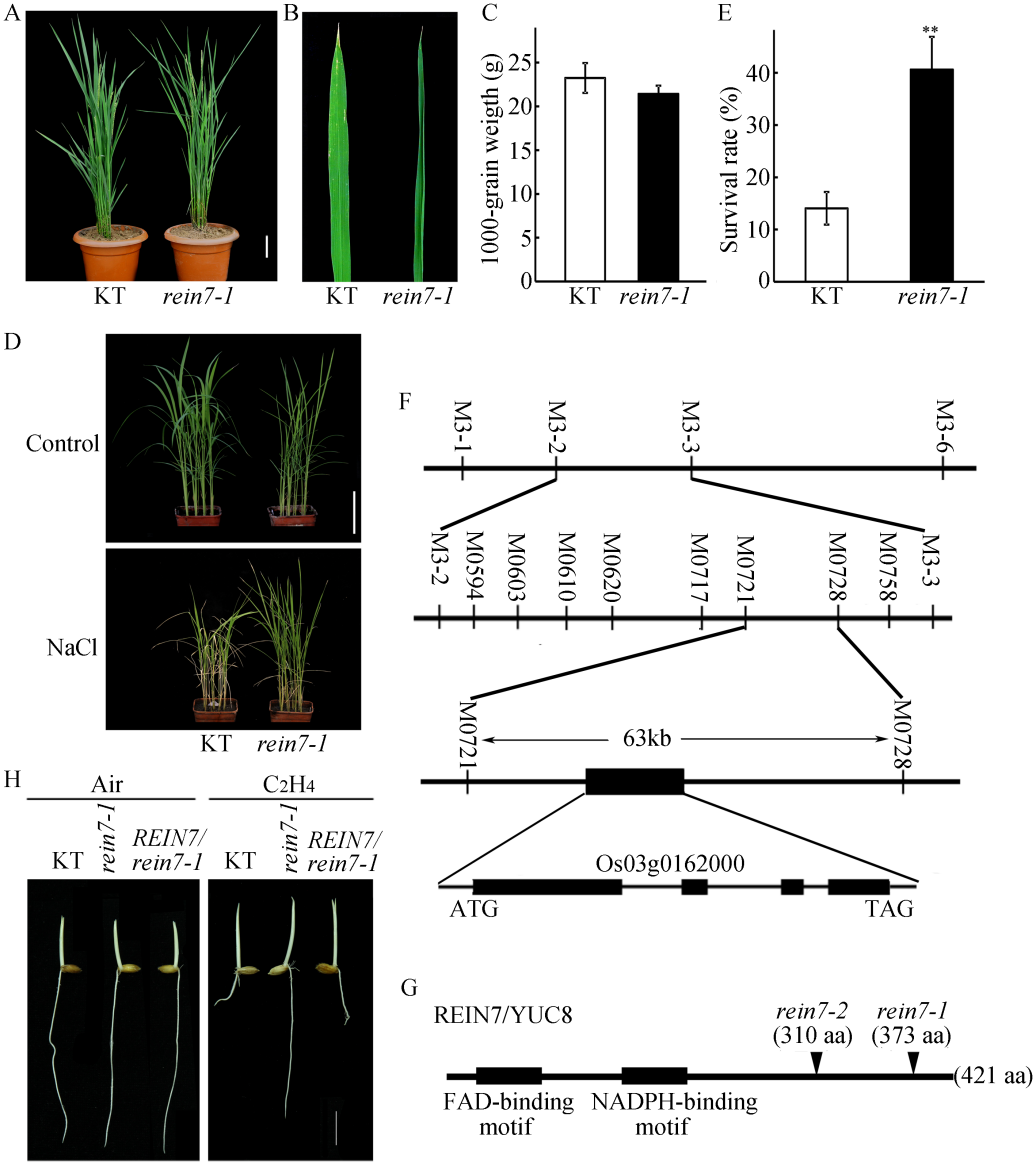
The *rein7-1*, a truncation of YUC8 at the C-terminus, displays rolled leaves and salt tolerance. (A) Plant morphology of Kitaake (KT) and *rein7-1* at the heading stage. Bar = 10 cm. (B) Typical image of rolled leaf. (C) The 1000-grain weight. Each value is the average of 30–50 plants. (D) Phenotypes of KT and *rein7-1* under salt stress. Control indicates that rice seedlings were grown under normal conditions, and NaCl indicates that seedlings were treated with 150 mM NaCl aqueous solution. Bar = 10 cm. (E) Survival rate after salt treatment in (D). Approximately 50–60 seedlings were used in each experiment. Bars indicate ± SD of three independent assays. ** indicates a significant difference compared to KT at *P* < 0.01. (F) Map-based cloning of the *YUC8*/*REIN7* gene. The locus was mapped to chromosome 3 within a 63 kb region between M0721 and M0728. ‘n’ indicates the number of samples used for map-based cloning, ‘M’ represents marker. (G) Mutation sites of two allelic mutants are indicated in the schematic diagram of the YUC8/REIN7 protein. (H) Functional complementation of the *rein7* mutant. The KT, *rein7-1* and complementary lines were treated with 10 ppm ethylene for 3 d under dark conditions. Bar = 10 mm.

**Fig 3:**
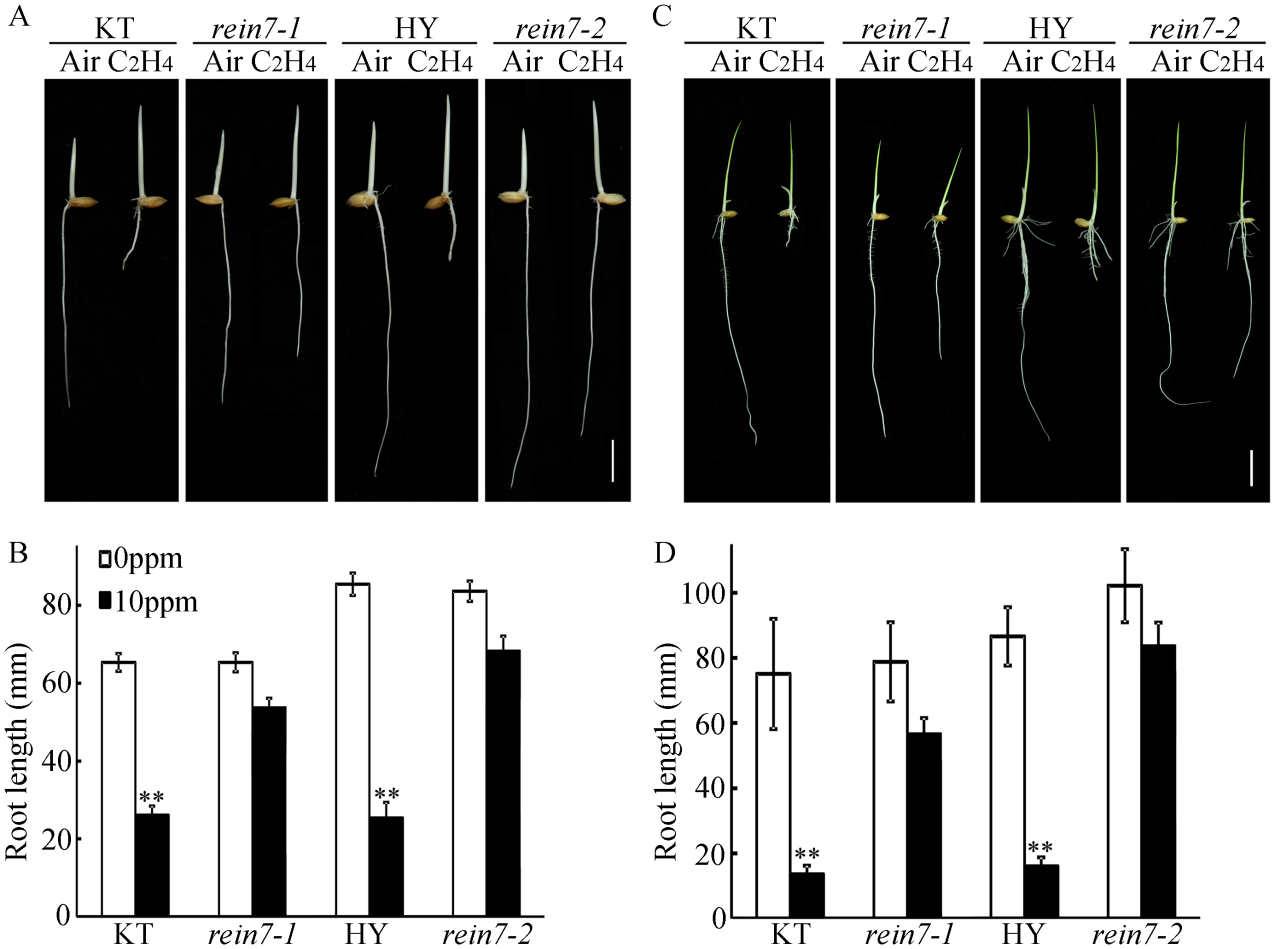
Mutation of YUC8/REIN7 confers an insensitive phenotype of primary root growth in response to ethylene. (A) and (C) Ethylene-response phenotypes of *rein7* in dark or in normal growth conditions. KT and Hwayoung (HY) are the wild types. Seedlings were grown in the dark or in normal growth conditions for 3 d in the absence (air) or presence of 10 ppm of ethylene. Bar = 10 mm. (B) and (D) Root length in (A) and (C), correspondingly. Each column is the average of 20–30 seedlings, and bars indicate ± SD. The experiment was repeated at least three times with similar results. ** indicates significant difference compared to air at *P* < 0.01.

The field-grown *rein7-1* mutant exhibited narrow, curled leaves, and enhanced salt tolerance but showed no significant difference in the 1000-grain weight compared with that of the wild-type plants (Fig 2A–2E), indicating that the *rein7-1* confers salt adaptation without compromising yield. Analysis with map-based cloning using the F_2_ population of *rein7-1* crossed with Dular (an *indica* cultivar) revealed that there is a single base pair substitution (G-A) in the fourth exon at nucleotide 2360 in Os03g0162000, resulting in a stop codon and loss of 47 amino acid residues from the C-terminal end of the REIN7 protein (Fig 2F–2G). The *rein7-2* mutant was previously reported with a phenotype of curled leaves [58]. Sequence analysis suggests that *REIN7* encodes a 421 amino acid protein containing two conserved sequence motifs, the FAD-binding motif and NADPH-binding motif (Fig 2G), and is a flavin-containing monooxygenase [59]. This protein shows significant sequence identity with YUC from *Arabidopsis*, which is required for the biosynthesis of auxin [18]. REIN7 corresponds to YUC8, belonging to the same group as rice YUC1 and *Arabidopsis* YUC1 and YUC4 (S5 Fig). To confirm that the mutation of YUC8/REIN7 locus is responsible for the mutant phenotype of *rein7-1*, we cloned a 5433 bp DNA fragment, including the complete Os03g0162000 genomic sequence, from Kitaake (a *japonica* cultivar) and transformed the construct into the *rein7-1* plants. Ethylene response assays showed that the altered ethylene responsiveness of *rein7-1* was rescued in the transgenic plants (Fig 2H), indicating that *YUC8/REIN7* is located at Os03g0162000 locus. To further analyze the ethylene response of the *rein7* mutants, we treated the two allelic mutants *rein7-1* and *rein7-2* with 10 ppm ethylene in dark or normal conditions; all of them exhibited reduced ethylene responses in roots in either dark or normal growth conditions (Fig 3A–3D). Thus our data showed that the C-terminal region of YUC8/REIN7 is required for ethylene-inhibited root elongation.

**Fig 4:**
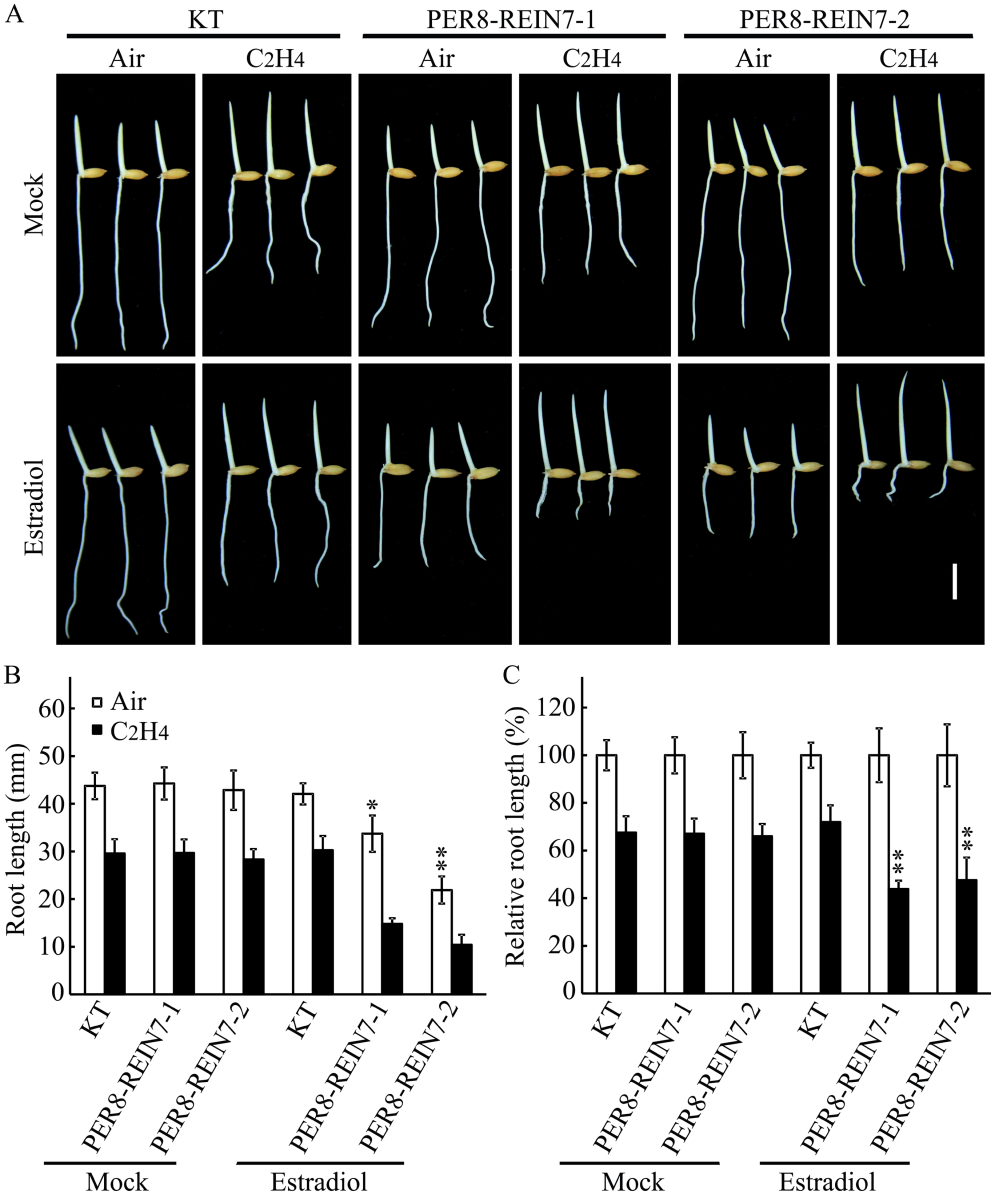
*YUC8*/*REIN7* overexpression enhances ethylene response in roots. (A) Ethylene response phenotypes of KT and inducible transgenic line (PER8-REIN7) seedlings grown in the dark for 3 d in the presence or absence of 2.5 μM estradiol. Bar = 10 mm. (B) Root length and (C) relative root length (ethylene-treated versus untreated in each genotype, respectively) in (A). Each column is the average of 20–30 seedlings, and bars indicate ± SD. The experiment was repeated at least three times with similar results. * and ** indicate significant difference compared to KT at *P* < 0.05 and *P* < 0.01.

To further study the function of *YUC8*/*REIN7* in rice ethylene response, we transformed the gene into the wild-type rice plants under the control of the CaMV 35S promoter. Due to alteration of the auxin content in the derived transgenic plants, most of the transformed *calli* exhibited overgrowth of the roots, and the rate of plant regeneration was quite low, and only two independent transgenic lines were obtained with a low transcription of *YUC8/REIN7* (S6B Fig). To more directly observe the gene function, we used an estradiol-inducible system for *YUC8*/*REIN7* expression. In the absence of estradiol treatment, the transgenic seedlings exhibited normal phenotypes and ethylene responses similar to those in the wild type (Fig 4A–4C). In contrast, when the plants were treated with estradiol, the primary root of transgenic seedlings was significantly shorter than that of the wild type. Following ethylene treatment, the transgenic seedlings showed enhanced ethylene response phenotypes in roots compared with those of the wild-type seedlings (Fig 4A–4C), further demonstrating that YUC8/REIN7 is required for ethylene-inhibited root elongation.

**Fig 5:**
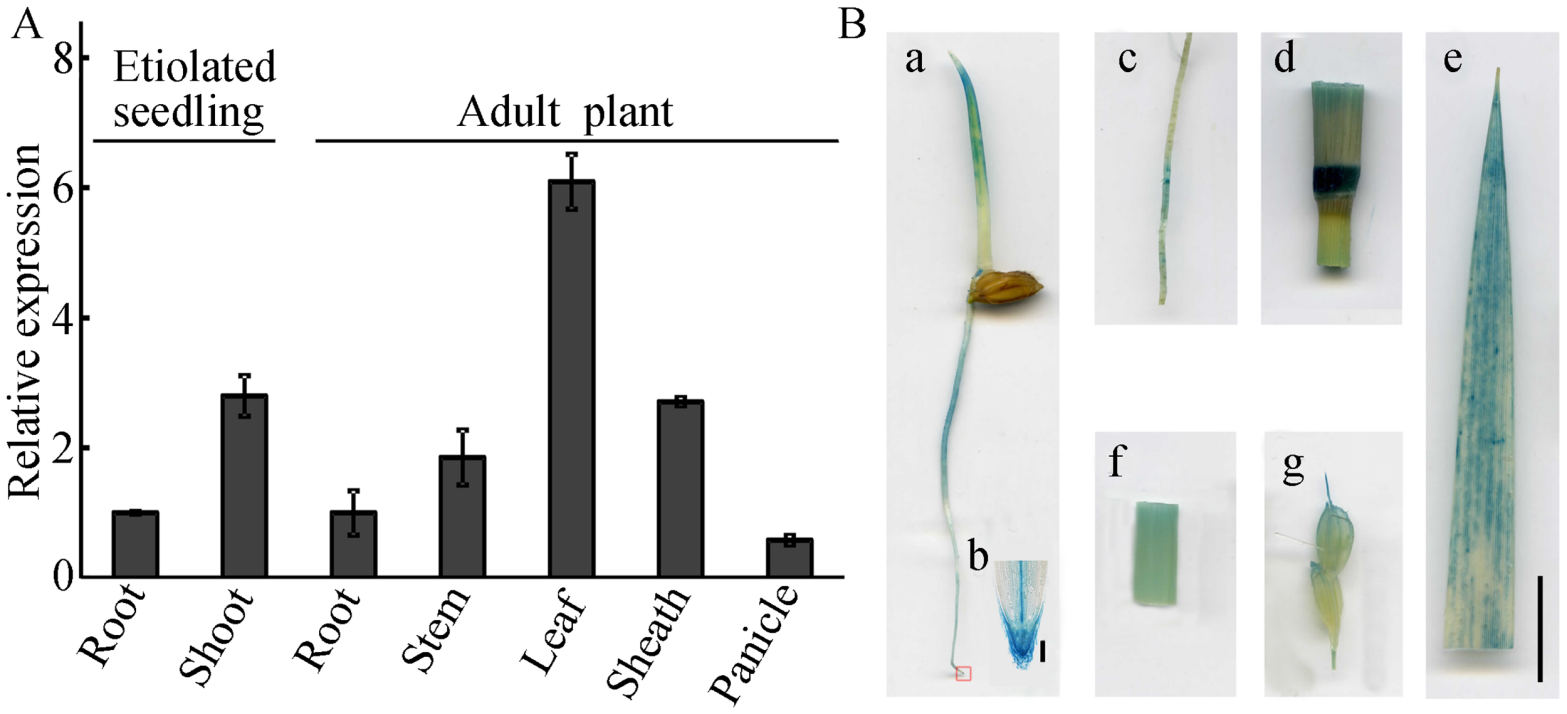
The transcripts of *YUC8/REIN7* are expressed in developmental tissues. (A) *YUC8/REIN7* expression in different rice tissues detected by qPCR. Bars indicate ± SD from five independent experiments. (B) Tissue-specific expression of *YUC8/REIN7* revealed by transgenic line (YUC8 promoter-GUS) analysis. (a) 3-d-old etiolated seedlings. (b) Root tip. Bar = 100 μm. (c) Root. (d) Young stem nodes. (e) Leaf. (f) Stem. (g) Developing grains. Bar = 10 mm.

To study the function of YUC8/REIN7 in rice growth and development, we investigated its expression patterns by qPCR. The transcripts were detected in all organs from vegetative to reproductive stages and were preferentially expressed in young leaves (Fig 5A). GUS staining driven by the *YUC8*/*REIN7* promoter in transgenic rice showed that the expression of *YUC8*/*REIN7* was located in roots and coleoptiles of etiolated seedlings (Fig 5Ba and 5Bb). In field-grown plants, *YUC8*/*REIN7* was expressed in the root tip, leaf, stem, young stem nodes and developing grains (Fig 5Bc–5Bg).

Next, we investigated whether YUC8/REIN7 was involved in auxin biosynthesis *in vivo*. We generated transgenic lines containing 35S:*REIN7* in the *Arabidopsis yuc1-1* mutant, which has defective auxin biosynthesis [60]. The REIN7-OX/*yuc1-1* plants exhibited longer hypocotyls, shorter primary roots and more root hairs than those of the control plants (S7A, S7D and S7E Fig). Mature leaves of REIN7-OX/*yuc1-1* were longer, narrower and curled downward compared with those of the wild type (S7B and S7C Fig). These phenotypes are similar to those caused by elevated auxin levels as previously reported [18], while REIN7m-OX/*yuc1-1* plants exhibited much longer petiole and slightly longer hypocotyl than the wild-type seedlings, but no obvious differences in roots and mature leaves compared with *yuc1-1* (S7 Fig), demonstrating that truncated YUC8/REIN7 is partial active. Considering that FAD and NADPH binding sites of the truncated YUC8/REIN7 are remained, it should be possible that the truncated protein still has partial activity to bind to FAD and NADPH, consistence with the incompletely insensitive phenotype in *rein7* mutants (Fig 3). These results suggest that YUC8/REIN7 is an auxin biosynthesis gene, and the C-terminus is important for its function.

**Fig 6:**
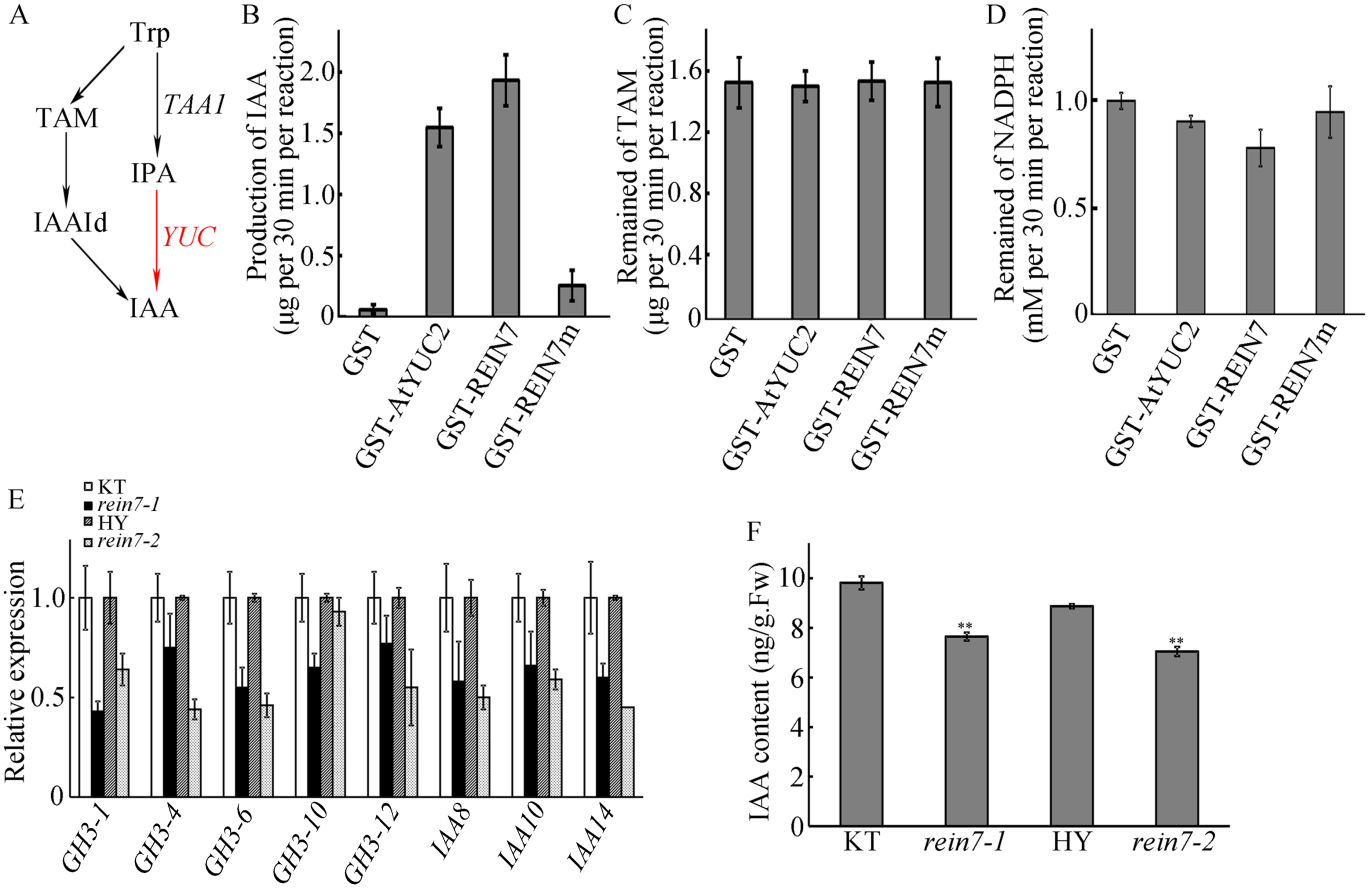
YUC8/REIN7 is mainly required for IPA-dependent auxin biosynthesis. (A) The simplified liner pathway of IAA biosynthesis. The red arrows indicate the function of YUC. Enzymatic assays with GST-AtYUC2, GST- REIN7, GST-REIN7m and the product IAA (B), the substrate TAM (C) were analyzed by LC-ESI-MS/MS. The bars represent ± SD from three independent experiments. (D) The content of remained NADPH in (C). The bars represent ± SD from three independent experiments. (E) Expression of auxin-inducible genes in 7-d-old normal grown seedlings. The bars represent ± SD from five independent experiments. (F) IAA content of 7-d-old normal grown seedlings. The bars represent ± SD from three independent experiments. ** indicates a significant difference compared to KT or HY at *P* < 0.01.

A previous study showed that *Arabidopsis* YUC1 converts tryptamine to N-hydroxylated tryptamine [18], but the mass spectrometry spectra were inconsistent with those for synthetic N-hydroxylated tryptamine [61]. Recent data showed that *yuc* mutants accumulate IPA, and *in vitro* data indicated that the YUC proteins convert IPA to IAA (Fig 6A) [15,16]. To determine whether YUC8/REIN7 functions in the conversion of IPA to IAA in the IPA pathway, we used kynurenine (Kyn), a potent inhibitor of *in vivo* TAA1/TAR activity [6], to treat the *YUC8*/*REIN7* transgenic lines. Our data showed that the short root phenotype of *YUC8*/*REIN7* overexpression lines was greatly suppressed by Kyn (S6A and S6C Fig). And this decrease of root growth suppressed by Kyn was further confirmed in estradiol-inducible transgenic lines (S6D and S6E Fig), suggesting that YUC8/REIN7 and TAA1/TAR work in a linear pathway to produce IAA. Next, we performed an enzymatic assay using purified GST-fused YUC8/REIN7 (S8A and S8B Fig). Our data showed that the GST-REIN7 fusion catalyzed the conversion of IPA to IAA. Loss of 47 amino acid residues from the C-terminal end of YUC8/REIN7 (GST-REIN7m) resulted in a significant decrease of enzymatic activity (Figs 6B, S9A and S9B), further supporting that the truncated protein of REIN7 is partially active. We did not detect the conversion of TAM in our assay conditions (Figs 6C, S9A and S9C), and the NADPH content in the reaction was slightly decreased when TAM was used as a substrate, indicating that there only have a few TAM turnovers (Fig 6D). These results suggest that YUC8/REIN7 mainly catalyzes conversion of IPA to IAA in the IPA pathway, and its C-terminus is important for enzymatic activity. Furthermore, the expression of auxin-responsive genes detected were decreased in *rein7* mutants (Fig 6E), consistent with the decreased IAA content in the lines in 7-d-old seedlings (Fig 6F). These results suggest that YUC8/REIN7 is an important factor required for auxin biosynthesis.

### Ethylene enhances the YUC8/REIN7-dependent auxin biosynthesis

**Fig 7:**
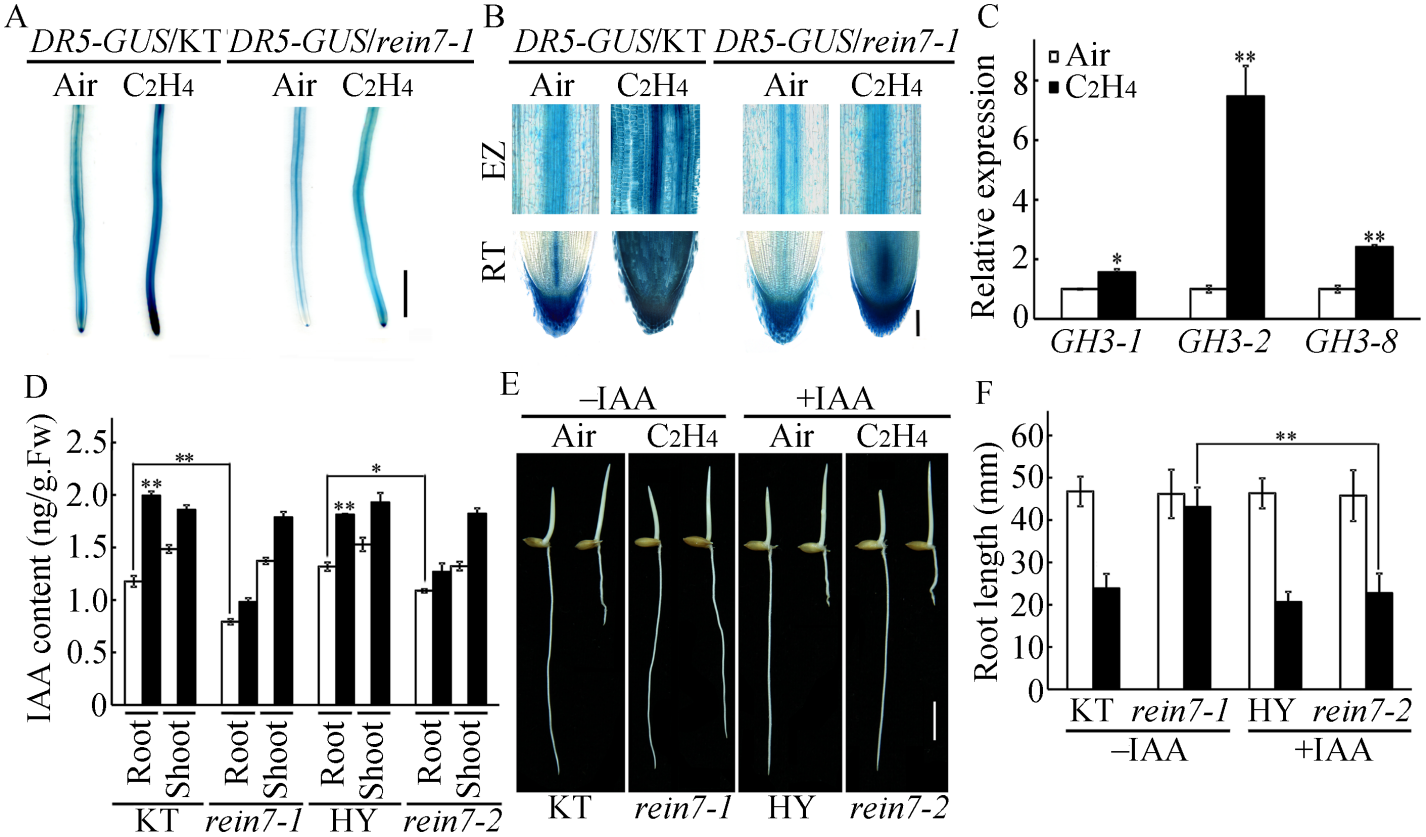
YUC8/REIN7-mediated auxin biosynthesis is required for ethylene-inhibited root growth in etiolated seedlings. (A) *DR5-GUS* expression in root. Seedlings of 3-d-old etiolated transgenic lines containing *DR5-GUS* in the wild type or the *rein7-1* background were treated with or without 10 ppm ethylene for 8 h before GUS activity was assayed. Bar = 10mm. (B) Root tip and elongation zone in (A). ‘RT’ represents root tip, ‘EZ’ represents elongation zone. Bar = 100 μm. (C) qPCR analysis of auxin-response gene expression in response to ethylene. Dark-grown 3-d-old wild-type seedlings were treated with 10 ppm ethylene for 3 h. The RNAs from roots were isolated for qPCR. (D) IAA levels in 3-d-old wild-type and *rein7* etiolated seedlings in the absence or presence of 10 ppm ethylene. (E) Rescue of the reduced ethylene sensitivity of *rein7-1* root by IAA. The wild-type and *rein7-1* seedlings were grown in the dark for 3 d in the absence or presence of 10 ppm ethylene, with or without supplementation of 10 nM IAA. Bar = 10 mm. (F) Quantification of root inhibition in (D). Each column is the average of 20–30 seedlings. The data are shown as the mean ± SD of three biological replicates. * and ** indicate significant differences between the compared two samples at *P* < 0.05 and *P* < 0.01, respectively.

YUC8/REIN7 participates in auxin biosynthesis, and multiple studies have demonstrated that ethylene enhances auxin biosynthesis to inhibit root elongation [9,32,33]. We then investigated whether YUC8/REIN7 is involved in ethylene-enhanced auxin biosynthesis in rice root. Thus, we introduced a *DR5*:*GUS* reporter, an auxin reporter responding to endogenous auxin [62], into the wild type and the *rein7* mutant. The expression of *DR5*:*GUS* in primary roots was significantly increased in the wild type after ethylene treatment, but this tendency was weakened in the *rein7* roots (Fig 7A and 7B). The expression of auxin-responsive genes was dramatically induced by ethylene in roots of the wild-type seedlings (Fig 7C). In addition, the endogenous IAA content in *rein7* was about 70% and 90% of that in the wild-type roots and shoots, respectively, indicating that YUC8/REIN7 mainly affects root auxin production. After ethylene treatment, endogenous IAA content did not significantly increased in *rein7* roots, although obviously enhanced in the wild-type roots (Fig 7D), demonstrating that ethylene-enhanced IAA production is predominantly inhibited in *rein7* roots. Thus, our data indicate that YUC8/REIN7 is essential for ethylene-enhanced auxin accumulation in rice roots.

Because the *YUC8/REIN7* mutation leads to the ethylene insensitivity in *rein7* roots, we next investigated whether addition of IAA could rescue the ethylene response of the mutant. We used 10 nM IAA in the complementation assay because this concentration of IAA had no obvious inhibitory effects on root growth in the wild-type seedlings (S10 Fig). In the presence of 10 nM IAA and 10 ppm ethylene, the defective response of *rein7-1* roots to ethylene was largely rescued (Fig 7E and 7F), suggesting that reduced ethylene sensitivity of *rein7* roots is most likely caused by the decrease of auxin biosynthesis.

**Fig 8:**
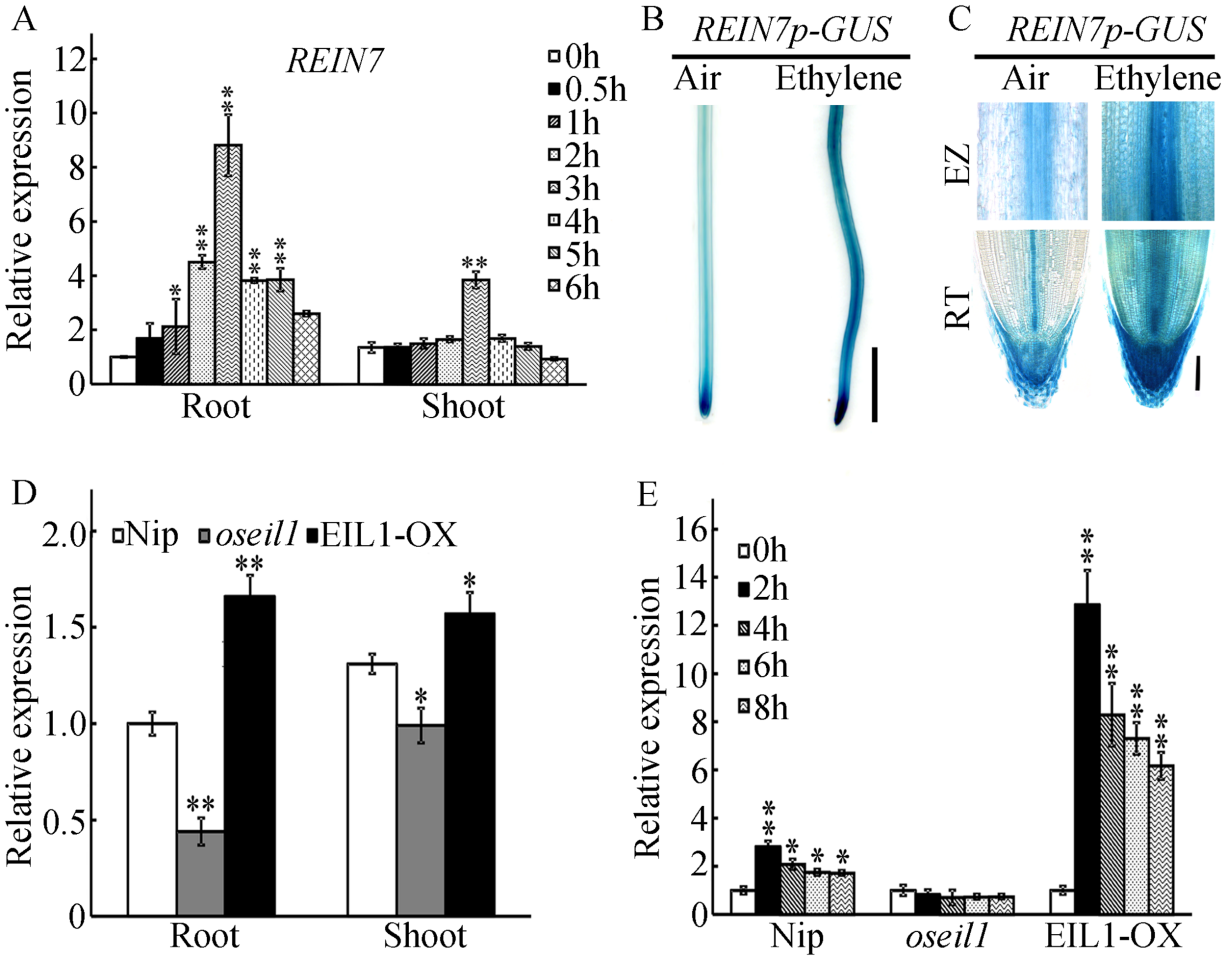
Mutation of OsEIL1 abolishes ethylene-induced *YUC8*/*REIN7* transcription. (A) Expression of *YUC8*/*REIN7* in response to ethylene. The wild-type seedlings were grown in the dark for 3 d and then treated with 10 ppm ethylene. The RNAs from roots and shoots were isolated and used for qPCR. (B) Ethylene-induced GUS activity in roots of transgenic plants harboring *REIN7p-GUS*. Etiolated seedlings of 3-d-old plants were treated with or without 10 ppm ethylene for 8 h before GUS activity was assayed. Bar = 10mm. (C) Root tip and elongation zone in (B). ‘RT’ represents root tip, ‘EZ’ represents elongation zone. Bar = 100 μm. (D) qPCR analysis of *YUC8*/*REIN7* expression in primary roots and shoots of Nip, *oseil1* and EIL1-OX seedlings grown in the dark for 3 d. (E) Expression of *YUC8*/*REIN7* in primary roots of Nip, *oseil1* and EIL1-OX seedlings grown in the dark for 3 d and then treated with 10 ppm ethylene. The data are shown as the mean ± SD of five biological replicates. * and ** indicate significant differences compared to 0 h at *P* < 0.05 and *P* < 0.01, respectively.

We then questioned whether ethylene activates *YUC8*/*REIN7* transcripts. Our qPCR analyses showed that *YUC8*/*REIN7* transcripts were significantly induced within 3 h with ethylene application in the wild-type roots and shoots (Fig 8A). Analysis with *YUC8*/*REIN7* promoter-GUS transgenic line also showed that ethylene treatment stimulated *YUC8/REIN7* promoter activity in roots (Fig 8B and 8C), suggesting a role for YUC8/REIN7 in root growth. Taken together, these findings reveal that ethylene transcriptionally activates the expression of *YUC8*/*REIN7*, resulting in auxin accumulation and inhibition of root elongation.

### YUC8/REIN7 functions downstream of OsEIL1 in ethylene-inhibited root elongation

Because ethylene-inhibited root elongation primarily mediates the ethylene signaling pathway, and ethylene transcriptionally activates the expression of *YUC8*/*REIN7*, we then examined whether the *YUC8*/*REIN7* transcripts were controlled by OsEIL1. Our qPCR analyses showed that the expression of *YUC8*/*REIN7* was significantly increased in *OsEIL1* overexpression seedlings but decreased in *oseil1* in both roots and shoots (Fig 8D), and ethylene-induced *YUC8*/*REIN7* expression was completely abolished in *oseil1* (Fig 8E). These results suggest that ethylene-inhibited root elongation *via* auxin biosynthesis is enhanced by OsEIL1 through regulation of *YUC8*/*REIN7* expression.

**Fig 9:**
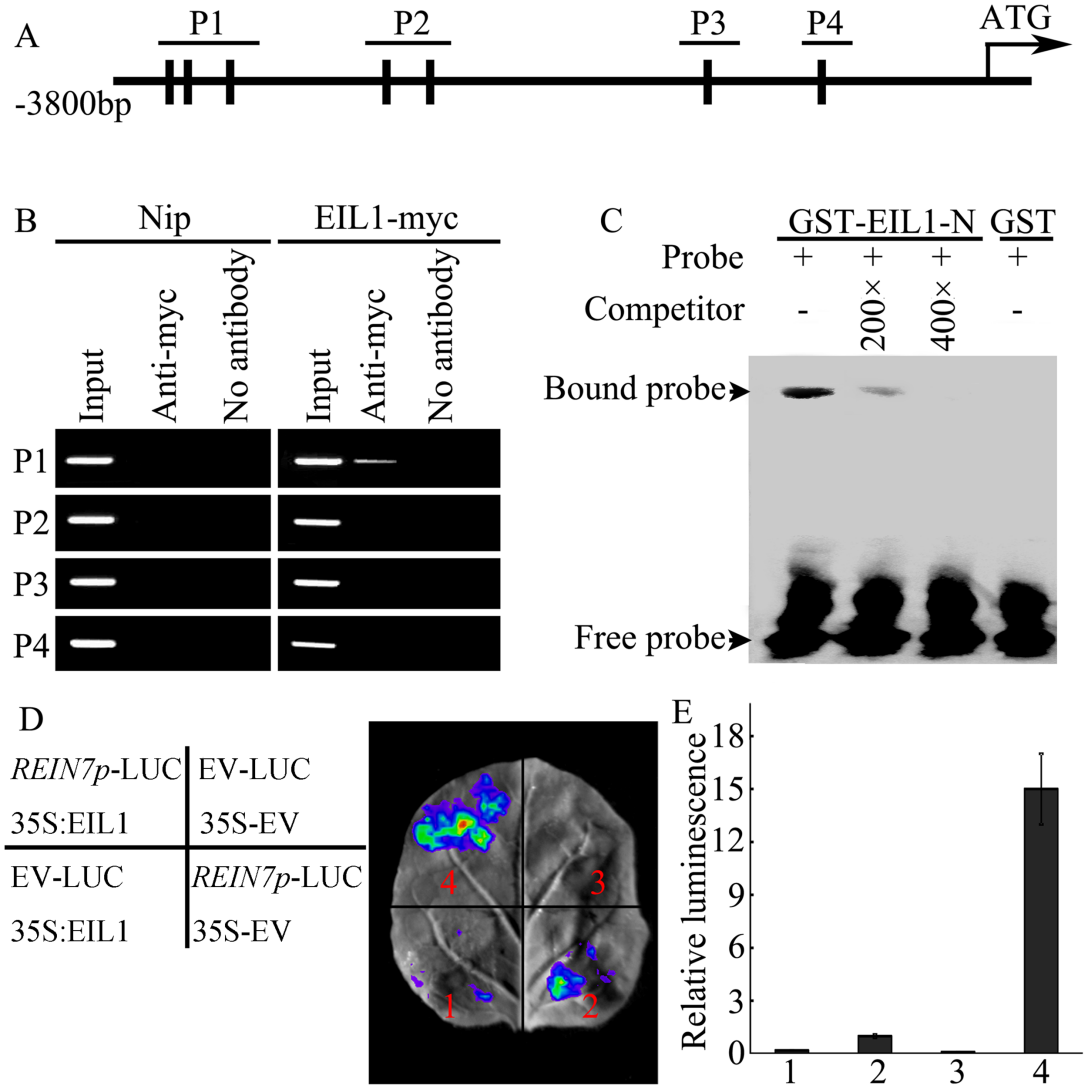
OsEIL1 directly binds to *YUC8/REIN7* promoter region. (A) Schematic diagrams of putative EIN3 binding site (EBS) in the promoter of *YCU8/REIN7*. Black boxes indicate the positions of the EBS. P1-P4 are fragments of the *YUC8/REIN7* promoter. (B) Anti-myc ChIP assays with DNA from 3-d-old etiolated seedling roots of Nip and overexpressing *OsEIL1* with myc-tag (EIL1-myc) transgenic plants. (C) EMSA assay for binding to EBS sequence in the promoter of *YUC8/REIN7* by OsEIL1 protein *in vitro*. Glutathione *S*-transferase (GST)-tagged OsEIL1 N-terminal fusion protein was incubated with biotin-labeled DNA fragments (Probe). Competition for the biotin-labeled promoter region was done by adding an excess of unlabeled probe (Competitor). Three biological replicates were performed with similar results. (D) The activation of OsEIL1 on the promoter activity of *YUC8/REIN7* by transient expression assay in tobacco leaves. ‘EV’ represents empty vector. Three biological replicates were performed with similar results. (E) Quantitative analysis of luminescence intensity for each comparison in (D).

To determine whether OsEIL1 functions as a direct regulator of *YUC8/REIN7*, we first analyzed the promoter sequence of *YUC8/REIN7* and found that there are seven EIN3-binding sites (EBS: ATGTA) (Fig 9A). Subsequently, we performed chromatin immunoprecipitation (ChIP) assay using the myc-tagged OsEIL1 (EIL1-myc) transgenic line. As shown in Fig 9B, the anti-myc antibodies precipitated the P1 fragment of the *YUC8/REIN7* promoter. Next, we conducted electrophoretic mobility shift assay (EMSA) with GST-EIL1-N fusion protein expressed in *E*. *coli*. As shown in Fig 9C, the GST-EIL1-N fusion protein was able to directly bind to the DNA probes containing the EBS motif as in the P1 fragment of *YUC8/REIN7* promoter, the binding was specific as demonstrated by competition assay using unlabeled competitor. These results indicate that OsEIL1 directly binds to *YUC8/REIN7* promoter *in vitro* and *in vivo*.

To test whether OsEIL1 could activate the expression of *YUC8/REIN7*, we used a tobacco transient expression assay system, the 3800 bp promoter sequence upstream from the ATG codon of *YUC8/REIN7* was fused to the *LUCIFERASE* (*LUC*) reporter gene and cotransfected with the effector plasmid harboring *35S*:*EIL1* into the tobacco leaves, the cotransfected effector significantly increased the LUC activity driven by *YUC8/REIN7* promoter, compared to control vector (Fig 9D and 9E). These results indicate that OsEIL1 could activate the expression of *YUC8/REIN7*.

**Fig 10:**
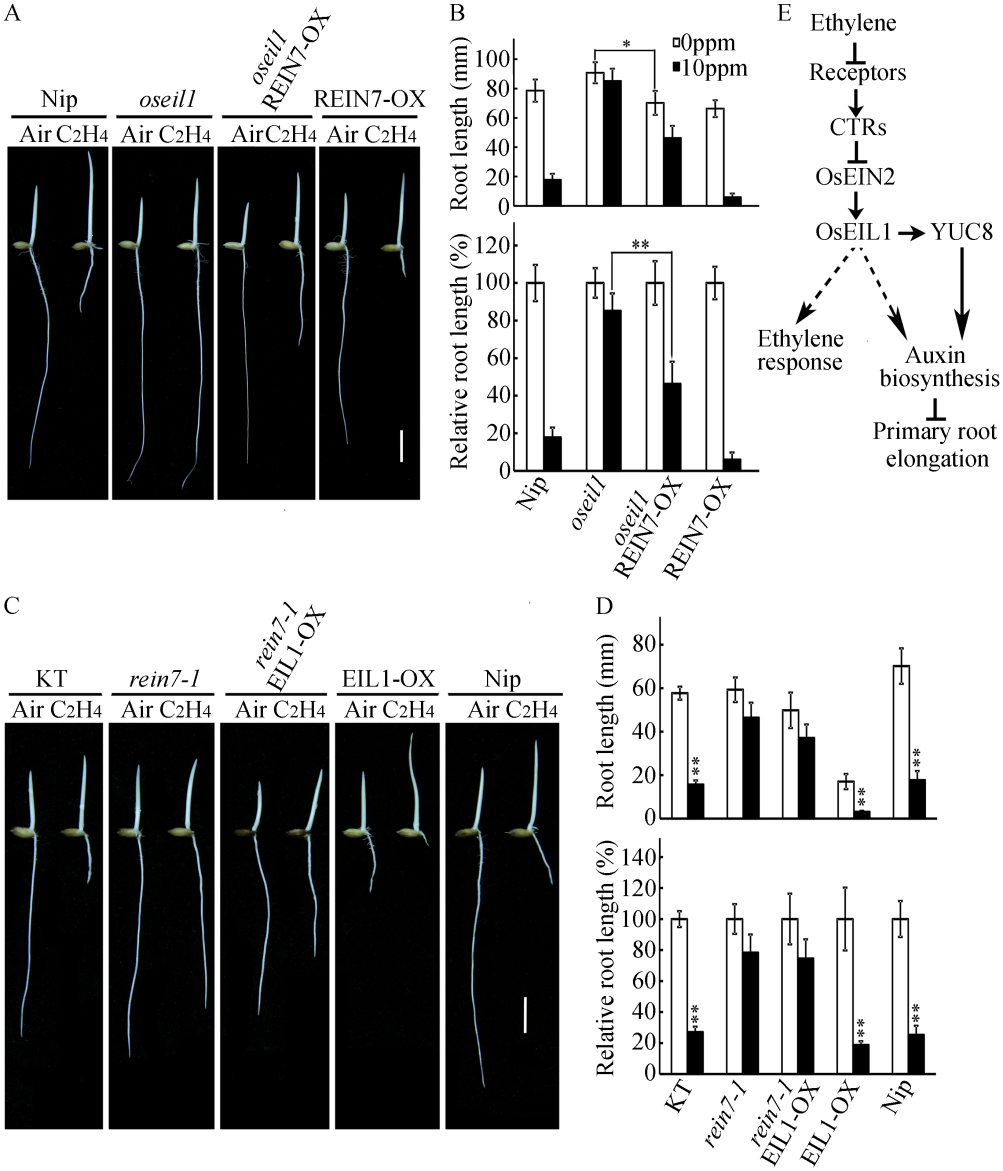
YUC8/REIN7 genetically functions downstream of OsEIL1. (A) Phenotypes of Nip, *oseil1*, *oseil1* REIN7-OX and REIN7-OX dark-grown seedlings in the presence or absence of 10 ppm ethylene for 3 d. Bars = 10 mm. (B) Root length and relative root length (ethylene-treated versus untreated in each genotype, respectively) in (A). (C) Phenotypes of KT, *rein7-1*, *rein7-1* EIL1-OX, EIL1-OX and Nip dark-grown seedlings in the presence or absence of 10 ppm ethylene for 3 d. Bars = 10 mm. (D) Root length and relative root length (ethylene-treated versus untreated in each genotype, respectively) in (C). In (B) and (D), each column is the average of 20–30 seedlings, and bars indicate ± SD. * and ** indicates a significant difference compared to air at *P* < 0.05 and *P* < 0.01. (E) A proposed model of ethylene-inhibited primary root elongation in rice. Ethylene signaling acts upstream of the auxin biosynthesis to regulate primary root elongation. YUC8/REIN7 is a key regulator required for ethylene-inhibited primary root elongation.

To examine the genetic relationship between YUC8/REIN7 and the ethylene signaling component OsEIL1, we first analyzed the ethylene response of the *oseil1* REIN7-OX plants that were obtained by overexpression of *YUC8*/*REIN7* on an *oseil1* background. Our observations revealed that the root length of *oseil1* REIN7-OX was shorter than that of *oseil1* but similar to that of REIN7-OX in the absent of ethylene. Upon exposure to ethylene, the root length of the *oseil1* REIN7-OX plants was significantly reduced compared with that of *oseil1* but was longer than that of REIN7-OX (Fig 10A and 10B). To test whether IAA could rescue the response of root elongation of *oseil1* to ethylene, we treated *oseil1* seedlings in the presence of 10 nM IAA and 10 ppm ethylene, the result showed that IAA partially rescued the response of root elongation of *oseil1* to ethylene (S11A and S11B Fig). These results suggest that there may have an OsEIL1-independent ethylene responsive pathway that regulates root elongation together with auxin, and YUC8/REIN7 can partially suppress ethylene insensitivity of roots in *oseil1*.

To confirm the above genetic relationship, we further generated *rein7-1* EIL1-OX plants by crossing the *rein7-1* mutant with EIL1-OX plants. Without ethylene treatment, the root length of *rein7-1* EIL1-OX seedlings was significantly longer than that of EIL1-OX seedlings. With ethylene treatment, the inhibition of root growth of EIL1-OX seedlings was partially alleviated in the *rein7-1* EIL1-OX seedlings (Fig 10C and 10D). Because YUC8/REIN7 plays an important role in ethylene-inhibited root elongation, and ethylene treatment increases the expression of *DR5*:*GUS* and IAA content in the *rein7-1* roots, these data are consistent with the detection of YUC gene expression in response to ethylene, indicating that YUC8/REIN7 is one of YUC members involved in ethylene-induced auxin accumulation in roots. Thus our data suggest that YUC8/REIN7 acts downstream of ethylene signaling pathway and the YUC8/REIN7-mediated pathway is partially required by OsEIL1 signaling for the regulation of the ethylene-inhibited root elongation.

## Discussion

Previous studies have shown that the interaction of ethylene and auxin affects multiple physiological processes, including root growth [7,19,33,34]. Rice roots grow in water-saturated environments during their life cycle, and this unique habit might confer different features of ethylene signaling between water-grown rice and dry-land-cultured *Arabidopsis* [44]. Although the crosstalk between ethylene and auxin has been extensively investigated in *Arabidopsis*, the roles of ethylene and auxin in rice root elongation are still unclear. In the present report, we showed that YUC8/REIN7 *via* IPA-dependent auxin biosynthesis is essential for ethylene-inhibited root elongation. This process could be enhanced by ethylene and was dependent on transcriptional modulation of OsEIL1. Thus, our findings reveal a mode of interplay between ethylene and auxin in rice root elongation, enhancing our understanding of ethylene signaling in rice.

Ethylene plays important roles in root development [5,7]. In *Arabidopsis*, ethylene inhibits root growth through the interaction with auxin. For example, auxin biosynthetic genes, such as *ASA1* [9,34] and *TAA1* [6,19], have been identified in this process. YUC family proteins are pivotal for auxin biosynthesis [13,18] and catalyze the conversion of IPA to IAA, a rate-limiting step in the IPA pathway [15,16]. However, the function of the YUC family in this process is not well understood. Considering the different ethylene response between *Arabidopsis* and rice [44], we investigated whether YUC family members are necessary for rice ethylene response. In this report, we showed that YUC family members, at least YUC5, YUC8 and YUC11, are important for ethylene-inhibited root elongation, implying that there is functional redundancy of YUC genes in this process. Furthermore, we found that the *YUC8*/*REIN7* mutation confers insensitivity to ethylene in root growth with reduced auxin levels, while *YUC8*/*REIN7* overexpression enhanced ethylene responses in roots, revealing that YUC8/REIN7 is an important factor in ethylene-inhibited root elongation.

Previous genetic studies demonstrated that YUC catalyzes the N-oxygenation of TAM [18], and recent reports showed that YUC has a role in the conversion of IPA to IAA in *Arabidopsis* [15,16]. Considering the distinct aspects between *Arabidopsis* and rice, we first biochemically showed that YUC8/REIN7 functions mainly through IPA-dependent IAA biosynthesis in rice. Moreover, transgenic *Arabidopsis* lines overexpressing *YUC8/REIN7* in the *Arabidopsis* mutant *yuc1-1* showed auxin overproduction phenotypes, which are consistent with the *AtYUC1* overexpression lines [18], suggesting that YUC8/REIN7 has similar functions to those of AtYUC1. In addition, the C-terminal region is important for YUC8/REIN7 enzymatic activity based on enzyme assay. Moreover, both of the mutants *rein7-1* and *rein7-2* exhibited reduced ethylene-insensitive in primary root growth, which FAD and NADPH binding sites are remained, implying that the truncated protein still has partial activity to bind to FAD and NADPH, supporting the observation of the incompletely insensitive phenotype in *rein7* mutants. Thus our results indicate that the YUC pathway is conserved in *Arabidopsis* and rice, YUC8/REIN7 regulates IAA production through IPA-dependent IAA biosynthesis in rice, and the C-terminus is important for YUC8/REIN7 function.

YUC proteins play an important role in IAA biosynthesis; however, recent studies in *Arabidopsis* indicated that AtYUC6 confers drought tolerance independently of auxin biosynthesis [63], indicating the diversity of YUC function. Consistent with the results, we found that mutation of *rein7-1* increased salt tolerance in this study. Most importantly, we discovered that YUC8/REIN7 mediates the ethylene-inhibited root growth as determined by analyses of root response to ethylene with *rein7* alleles. Because the *YUC8*/*REIN7* mutation decreases the auxin content, possibly through disruption of auxin biosynthesis, we propose that auxin may be a factor participating in ethylene-inhibited root growth. This conclusion is strongly supported by the following evidence: (1) ethylene treatment enhanced the *DR5-GUS* signal in roots; (2) ethylene induced *YUC8*/*REIN7* expression and auxin accumulation specifically in roots; (3) exogenous application of IAA largely recovered the defective response of *rein7* roots to ethylene; (4) ethylene induced expression of various auxin-responsive genes; (5) *YUC8*/*REIN7* overexpression resulted in enhanced ethylene response in roots; and (6) *YUC8*/*REIN7* function is a key factor required for the ethylene signaling component *OsEIL1*-mediated root ethylene response. Thus, ethylene-inhibited root growth requires YUC8/REIN7-dependent auxin biosynthesis.

EIN3 acts as a positive regulator at the most downstream position of the ethylene signaling transduction pathway and constitutes a major fraction of the ethylene response [30]. In rice, there are six members in the small family of the EIN3-like homologues (OsEIL1 to OsEIL6). Among these, OsEIL1 and OsEIL2 show the highest similarity to *Arabidopsis* EIN3 and spatially regulate ethylene response of the roots and coleoptiles in etiolated seedlings [44,50]. Because of the *rein7* ethylene response in roots, the relationship between YUC8/REIN7 and the ethylene signaling component OsEIL1 was genetically identified, i.e., ethylene induces *YUC8/REIN7* expression depending on OsEIL1, and OsEIL1 directly binds to YUC8/REIN7 promoter and positively regulates its expression, revealing that YUC8/REIN7 is key factor required for ethylene-inhibited root growth and acts downstream of ethylene signaling. Moreover, overexpression of *YUC8/REIN7* in an *oseil1* background partially rescued the ethylene response in *oseil1* mutant, consistent with the detection that YUC8/REIN7 is one of YUC members involved in ethylene-induced auxin accumulation in roots, indicating that the YUC8/REIN7-mediated pathway is partially required by OsEIL1 signaling for the regulation of the ethylene-inhibited root elongation. Furthermore, the observation that supplementation of IAA partially rescues the response of root elongation of *oseil1* to ethylene implies that there might have an OsEIL1-independent ethylene responsive pathway that regulates root elongation together with auxin.

In *Arabidopsis*, ABA-inhibited root growth is dependent on ethylene biosynthesis [53]. Our previous studies have shown that ABA represses ethylene biosynthesis through ABSCISIC ACID INSENSITIVE 4 (ABI4)-mediated transcriptional repression of *ACS4* and *ACS8* in *Arabidopsis* [54,55]. Moreover, studies in *Arabidopsis* have established that ethylene inhibits root growth through auxin action by modulating its biosynthesis, transport and signaling [19,32–35,37,38]. Additionally, various auxin biosynthesis genes were directly regulated by ethylene signaling components [9,40], strongly suggesting that ABA may exert its effect on root inhibition through regulating ethylene biosynthesis to stimulate accumulation of auxin in roots. These findings are different from previous reports, indicating that ethylene inhibits root growth by regulating accumulation of ABA [48,52]. All these findings suggest that different mechanisms are present in rice. Our present results demonstrated that ethylene inhibits root growth largely through auxin function, and YUC play an important role in this process. However, the crosstalk between auxin and ABA in rice is still unclear. ABA may regulate root elongation through the auxin pathway or vice versa, or the two hormones might act independently to mediate ethylene response. Our previous study indicated that ERF2 is required for the control of rice root architecture, ABA and ethylene response by fine-tuning the expression of genes involved in hormone signaling pathways [64]. Further investigations of the relationships among these hormones should elucidate their interaction in the control of rice roots.

Taken together, our results in the present investigation support a model (Fig 10E) that ethylene stimulates auxin biosynthesis in roots through the ethylene signaling component OsEIL1. YUC8/REIN7 is one of the factors that modulate auxin biosynthesis. And this regulation of *YUC8/REIN7* is directly activated by OsEIL1. As a consequence of activating *YUC8/REIN7* expression, ethylene increases the accumulation of auxin, which in turn decreases root elongation.

## Materials and methods

### Plant materials and grown conditions

The *Arabidopsis yuc1-1* mutant used in this study was the SALK-106293 line as previously reported [60]. The rice knockout mutants *osein2*, *oseil1*, and overexpressing *OsEIN2* (EIN2-OX) or *OsEIL1* (EIL1-OX) transgenic lines were previously identified [43,50]. The isolation of ethylene-response *rein* mutants and ethylene treatment were performed as previously described [43]. The *YUC8* (Os03g0162000) T-DNA knockout mutant *rein7-2* (PFG_1C-07050.R) is on a Hwayoung (HY) background, identified by PCR using the T-DNA right border primer RB (5'-CCACAGTTTTCGCGATCCAGACTG-3') and gene-specific primers flanking the insertion site (RP, 5'-ATTCTGGCATGGAAGTGAGC-3'), was obtained from the POSTECH Biotech Center [65].

For the salt-tolerance assays in rice seedlings, the germinated rice seeds were cultured in soil for 2 weeks under normal growth conditions, watered with a 150 mM NaCl solution, and cultured for another 10–15 days before the phenotypes were observed. For material propagation, crossing, and investigating agronomic traits, rice plants were cultivated at the Experimental Station of the Chinese Academy of Agricultural Sciences in Beijing during the natural growing seasons.

### Treatments and analysis of root growth

IAA, yucasin, and Kyn treatments were performed as previously described [52]. Briefly, germinated rice seeds were placed on cheesecloth on a stainless steel sieve that was placed in an air-tight plastic box of 10 L volume and incubated at 28°C. The seeds were subjected to the treatment with 4 L of water containing either 10 nM IAA, 10 μM yucasin/Kyn, or 10 ppm ethylene gas. The ethylene treatment was performed as previously described [43]. IAA was dissolved in ethanol, yucasin and Kyn were in DMSO. The controls were conducted with treatments containing equivalent volumes of air, ethanol or DMSO. At the end of the period, the roots were scanned and their length was measured from digitized images using Image J software.

### Map-based cloning of the *REIN7* gene

F_2_ mapping populations were generated from crosses between the *rein7-1* mutant and *indica* variety Dular. Genomic DNA was isolated from seedlings with mutant phenotypes. A total of 246 mutant individuals selected from the F_2_ populations were used for fine mapping. PCR-based markers were developed based on the sequence difference between the *japonica* variety Nipponbare and *indica* variety 9311 (http://www.gramene.org/resources/). The primer sequences of the molecular markers used are listed in S1 Table. The *REIN7* locus was mapped to chromosome 3 between M0721 and M0728 in a 63 kb region that contains 7 genes. The candidate gene was finally determined by DNA sequencing of all the candidate genes within this region.

### Quantitative real-time PCR (qPCR)

Total RNA was extracted from an approximately 0.5 g sample from 3-d-old seedlings or young leaves with an Ultrapure RNA Kit (CWBIO, CW0581S) according to the manufacturer’s instructions. Total RNA (approximately 2 μg) from each sample was reverse transcribed to cDNA with HiScript II Q RT SuperMix for the qPCR reaction, according to the manufacturer’s instructions (Vazyme, R223-01). qPCR was performed according to the manufacturer’s instructions (Bio-Rad iQ5), as previously described [66]. The rice *Actin1* gene was used as the internal standard to normalize gene expression. The qPCR primers are listed in S1 Table.

### Generation of transgenic rice

For overexpression of *OsEIL1* in rice, the full coding sequence of *OsEIL1* was cloned into the plant expression vector pCAMBIA1307 using the *Xba* I and *Bam*H I sites. For overexpression of *YUC8/REIN7* in rice, the full coding sequence of *YUC8/REIN7* was cloned into the plant expression vector pCAMBIA1307 using the *Sal* I and *Bam*H I sites. To generate *YUC8/REIN7* inducible transgenic plants with an estradiol-inducible promoter, the full coding sequence of *YUC8*/*REIN7* was cloned into the vector pER8 using the *Csp451* and *Spe* I sites. For complementation, the *YUC8*/*REIN7* genomic sequence (2504 bp), the upstream 2395 bp sequence of the *YUC8*/*REIN7* ATG, and the downstream 534 bp sequence of the *YUC8*/*REIN7* TGA were used. The full sequence (5433 bp) was cloned into the pCAMBIA1300 vector using the *Bam*H I and *Kpn* I sites through In-Fusion cloning technology. For generation of the *YUC8*/*REIN7p*-GUS construct, the 4365 bp promoter region upstream of the start codon of *YUC8*/*REIN7* was cloned into the pCAMBIA1381Z vector using the *Xma* I and *Sal* I sites. All vectors were introduced into *Agrobacterium tumefaciens* strain EHA105 through electroporation, and the resulting strains were introduced into the rice variety Nipponbare or Kitaake. The primers used for the constructs are listed in S1 Table.

### Histochemical staining of GUS

For the histochemical staining of GUS in transgenic rice, the samples were incubated in sodium phosphate buffer (pH 7.0) containing 0.1% vol/vol Triton X-100 and 2 mM X-Gluc at 37°C for 12 h. After the samples were rinsed with 70% ethanol until the tissue cleared, they were photographed. To produce transverse section of roots, root segments were embedded in 3% agar. Transverse sections (25 μm) of root were produced using a vibratome (Leica VT 1000 S). The images of rice root autofluorescence were taken under a microscope (Nikon ECLIPSE Ni).

### The expression and purification of YUC8 protein *in vitro*

The coding sequences of *YUC8*/*REIN7* and truncated *YUC8*/*REIN7* (loss of 47 amino acid residues, *YUC8*/*REIN7m*) were amplified by PCR using the primers described in S1 Table and then linked to the T-vector for sequencing. The correct *YUC8*/*REIN7* and *YUC8*/*REIN7m* coding sequences were cloned into the expression vector pGEX-6p-1 using the *Bam*H I and *Sal I* sites and fused with a GST-tag. Next, the constructed vectors were transformed into the *E*. *coli* BL21 (DE3) strain, and the transformed strains were cultured in Luria–Bertani (LB) medium and harvested after induction with 1 mM isopropyl β-D-1-thiogalactopyranoside (IPTG) at 16°C for 8 h. The fusion proteins were extracted with the lysis buffer containing 50 mM Tris-HCl (pH 8.0), 500 mM NaCl, and 1% (vol/vol) Tween 20, and 20% (wt/vol) glycerol. The recombinant proteins were purified using a *ProteinIso* GST Resin according to the manufacturer’s instructions (Transgen, DP201-01). The concentration of the purified recombinant proteins was determined using the bicinchoninic acid protein assay (CWBIO, CW0014). The components of the purified proteins were analyzed by SDS-PAGE electrophoresis and Western blotting. The purified protein was immediately divided into aliquots and frozen in liquid nitrogen, and stored at -80°C for the further experiments.

### Enzyme assay

The enzyme assays of GST-YUC8/REIN7 and GST-YUC8/REIN7m were performed as described [15]. Briefly, 20 μg of purified YUC8/REIN7, YUC8/REIN7m or AtYUC2 protein were added to a 100 μL reaction system containing 100 μM IPA or TAM, 40 μM FAD, and 1 mM NADPH in PBS buffer (pH 7.4) and incubated at 30°C for 30 min. IPA/TAM was added to the reaction just before the incubation at 30°C. In order to reduce the non-enzymatic conversion of IPA, the enzyme reactions were stopped by addition of acetonitrile and snap freezing in liquid nitrogen. And then, 20 μL of the mixture was injected into the HPLC instrument (Shimadzu LC-10A), and chromatographic separation was achieved on an YMC-Triart Diol-HILIC column (4.6 × 250 mm, 5 μm; YMC) and detected at 254 nm with an SPD M10A detector. The samples were eluted at a flow rate of 0.8 mL/min with 0.8% acetic acid (solvent A) and 100% acetonitrile (solvent B). Fractions eluting TAM (4.2 min)/IAA (7.2 min) were collected and further analyzed by LC-ESI-MS/MS in National Centre for Plant Gene Research (Beijing). The measurement meathod of TAM was similar to IAA. In parallel, 20 μg GST were used as a control, and only small amounts of IAA were produced non-enzymatically from IPA in a control reaction. The NADPH content was determined by measuring the optical density at 340 nm using a spectrophotometer.

### IAA content measurement

IAA was quantified as previously described [6]. Briefly, 200 mg (fresh weight) of whole root or shoot for each treatment was quickly frozen in liquid nitrogen and ground into a fine powder, and then, tissues were homogenized and extracted for 24 h in methanol containing ^2^H-IAA (CDN isotopes) as an internal standard. Purification was performed using an Oasis Max solid phase extract cartridge (Waters) after centrifugation. IAA measurement was carried out with a liquid chromatography–tandem mass spectrometry system consisting of Acquity Ultra Performance Liquid Chromatography (Acquity UPLC; Waters) and a triple quadruple tandem mass spectrometer (QTRAP 5500; AB SCIEX).

### ChIP-PCR assay

ChIP was conducted as described [67]. Briefly, approximately 2 g sample from 3-d-old overexpressing *OsEIL1* with myc-tag (EIL1-myc) and Nipponbare etiolated seedling root were harvested and fixed with 1% formaldehyde in PBS under vacuum for 30 min at room temperature. After three washes with sterile deionized water, the samples were ground to a fine powder to extract the proteins and DNAs. The chromatin solution was then sonicated to shear the DNA into fragments. After centrifuging, the chromatin pellet was re-suspended in 300 μL buffer containing 50 mM Tris-HCl (pH 8.0), 10 mM EDTA, 1% SDS, 1 mM PMSF (phenylmethanesulfonyl fluoride) and protease inhibitors. The above solution was divided into three portions and then 900 mL buffer [1.1% Triton X-100, 1.2 mM EDTA, 16.7 mM Tris-HCl (pH 8.0) and 167 mM NaCl] was added. After adding 40 μL of salmon sperm DNA to each chromatin sample with gentle rotation and overnight incubation at 4°C, the pellet was rinsed three times and re-suspended in a buffer containing 50 mM Na_3_PO_4_ (pH 8.0), 167 mM NaCl, 10 mM imidazole and protease inhibitors. The input solution was then used for performing immunoprecipitation using anti-myc antibodies. The DNA fragments were cleaned up using a PCR DNA purification kit (Tiangen, DP214). Promoter fragments were amplified using 1 μL of purified DNA as a template in each PCR reaction. Primer sequences for ChIP PCR experiments are provided in S1 Table.

### Electrophoretic mobility shift assay (EMSA)

To construct plasmid for the expression of N-terminal OsEIL1 (amino acids 1–350) region in *E*. *coli* BL21 (DE3), DNA fragment corresponding to the region was obtained and inserted into the pGEX-6p-1 vector using the *Bam*H I and *Not* I sites.

EMSA was performed as previously described [50]. Briefly, single-stranded complementary oligonucleotide fragments corresponding to region of *YUC8/REIN7* promoter (S1 Table) harboring the EBS elements were synthesized and biotinylated using the Biotin 3’ End DNA Labeling Kit (Thermo Fisher Scientific, 89818). Biotinylated and unlabeled complementary oligonucleotide pairs were annealed to make double-stranded biotin-labeled probes and competitors by mixing together equal amounts, denatured at 90°C for 1 min, then slowly cooled. EMSA reaction solutions were prepared according to the manufacturer’s protocol (LightShift Chemiluminescent EMSA Kit; Thermo Fisher Scientific, 20148). Reaction solutions were incubated for 20 min at room temperature. The protein-probe mixture was separated on a 5% polyacrylamide native gel and transferred to a nylon membrane (GE). After UV light cross-linking, the DNA on the membrane was detected using the Chemiluminescent Nucleic Acid Detection Module (Thermo Fisher Scientific, 89880).

### Transactivation assay in tobacco leaves

Transactivation assay was performed as previously described [50]. Briefly, the 3.8 kb sequence upstream from the ATG codons of *YUC8/REIN7* was inserted into pGWB35 to generate promoter:LUC reporter construct using Gateway technology (Invitrogen). The reporter plasmid and the construct containing 35S:EIL1 were transformed into *A*. *tumefaciens* strain GV3101. The strains were incubated in Luria-Bertani medium and finally resuspended in infiltration buffer (10 mM MES, 0.2 mM acetosyringone, and 10 mM MgCl_2_) to an ultimate concentration of optical density at OD_600_ = 1. Equal amounts of different combined bacterial suspensions were infiltrated into the young leaves of 5-week-old tobacco (*Nicotiana tabacum*) plants using a needleless syringe. After infiltration, the plants were grown first in the dark for 12 h and then kept with 16 h of light/8 h of dark for 48 h at 24°C before CCD imaging. The leaves were sprayed with 100 mM luciferin (Promega) and placed in the dark for 5 min. LUC activity was observed with a low-light cooled CCD imaging apparatus (iXon; Andor Technology). Experiments were performed with three independent biological replicates.

### Accession numbers

Sequence data from this article can be found in the MSU7.0 database (http://rice.plantbiology.msu.edu/) under the following accession numbers: *OsActin1*, Os03g50885; *GH3-1*, Os01g57610; *GH3-2*, Os01g55940; *GH3-4*, Os05g42150; *GH3-6*, Os05g05180; *GH3-8*, Os07g40290; *GH3-10*, Os07g38860; *GH3-12*, Os11g08340; *IAA8*, Os02g49160; *IAA10*, Os02g57250; *IAA14*, Os03g58350.

## Supporting information

S1 Fig Ethylene-inhibited primary root growth is mediated by ethylene signaling. (A) Root and coleoptile phenotypes of *japonica* (Nip, HY, KT) and *indica* (IR29, YD6-Yangdao #6, ZS97-Zhenshan 97) cultivars treated with air, 10 ppm ethylene, 1 ppm 1-MCP, 1 ppm 1-MCP plus 10 ppm ethylene, 0.2 μM AVG, and 0.2 μM AVG plus 10 ppm ethylene. Rice seedlings were grown in the dark for 3 d in the presence of various reagents. Bar = 10 mm. (B) Root length for the plants shown in (A). (C) Coleoptile length for the plants shown in (A). Values are shown as the mean ± SD of 20–30 seedlings per genotype. The experiment was repeated at least three times with similar results.
(TIF)

S2 Fig Disabled-ethylene signaling disturbs ethylene responses in roots and coleoptiles. (A) Ethylene-response phenotypes of Nip, *osein2*, EIN2-OX, *oseil1* and EIL1-OX seedlings. The etiolated seedlings were grown in air or 10 ppm ethylene for 3 d. Bar = 10 mm. (B) Root length for the plants shown in (A). (C) Coleoptile length for the plants shown in (A). Values are shown as the mean ± SD of 20–30 seedlings per genotype. The experiment was repeated at least three times with similar results.
(TIF)

S3 Fig Ethylene regulates transcription of YUC genes. Nip, *osein2*, EIN2-OX, *oseil1* and EIL1-OX seedlings grown in the dark for 3 d and then treated with or without 10 ppm ethylene for 3 h. The RNAs from roots were isolated and used for qPCR. The experiment was repeated at least five times with similar results. ‘ND’ represents not detected. Bars indicate ± SD.
(TIF)

S4 Fig *rein* mutants display ethylene-response phenotypes. (A) Ethylene-response phenotypes of various *rein* mutants. The etiolated seedlings were grown in air or 10 ppm ethylene for 3 d. Bar = 10 mm. (B) Root length of the wild type and *rein* mutants in response to ethylene. (C) Coleoptile length of the wild type and *rein* mutants in response to ethylene. Each column is the average of 20–30 seedlings, and bars indicate ± SD.
(TIF)

S5 Fig Phylogenetic analysis of YUC8/REIN7 and its homologous proteins from other plants. The phylogenetic tree of 14 rice and 11 *Arabidopsis* YUC genes was constructed using DNAMAN. Bootstrap analysis values are shown at the nodal branches.
(TIF)

S6 Fig Kyn reduces the auxin phenotypes of YUC8/REIN7 overexpression lines. (A) Root phenotype of Nip, constitutive overexpressing YUC8 transgenic (OX-1 and OX-2) etiolated seedlings. The seedlings were grown in the dark for 3 d in the presence or absence of 10 μM Kyn. Bar = 10 mm. (B) *YUC8/REIN7* expression in 3-d-old etiolated seedlings. The experiment was repeated at least five times with similar results. Bars indicate ± SD. (C) Root length in (A). (D) Root phenotype of KT and inducible transgenic (PER8-REIN7) etiolated seedlings. The seedlings were grown in the dark for 3 d in the presence or absence of 2.5 μM estradiol, with or without supplementation of 10 μM Kyn. Bar = 10 mm. (E) Root length in (D). In C and E, each column is the average of 20–30 seedlings, and bars indicate ± SD. * and ** indicates a significant difference compared to mock at *P* < 0.05 and *P* < 0.01.
(TIF)

S7 Fig YUC8/REIN7 complements the function of *Arabidopsis* AtYUC1 in IPA-dependent auxin biosynthesis. (A) The seedling phenotypes of Col-0, *yuc1-1*, transgenic lines overexpressing truncated (REIN7m-OX/*yuc1-1*) or full-length YUC8/REIN7 (REIN7-OX/*yuc1-1*) in *Arabidopsis yuc1-1* mutant grown on MS medium for 7 d. (B) The phenotypes of adult Col-0, *yuc1-1*, REIN7m-OX/*yuc1-1* and REIN7-OX/*yuc1-1* lines. (C) Mature leaves of the Col-0, *yuc1-1*, REIN7m-OX/*yuc1-1* and REIN7-OX/*yuc1-1* lines. (D) Root length in (A). (E) Hypocotyl length in (A). Each column is the average of 20–30 seedlings and bars indicate ± SD. * and ** indicate significant differences compared to *yuc1-1* at *P* <0.05 and *P* < 0.01, respectively.
(TIF)

S8 Fig The purified recombination protein was determined by SDS-PAGE electrophoresis and Western-blotting. (A) The purified proteins of GST-REIN7 and GST-REIN7m expressed in *E*. *coli* were identified by SDS-PAGE electrophoresis.–IPTG and +IPTG: the total proteins from *E*. *coli* that were induced or not by IPTG, respectively; Purified: the purified recombined protein. (B) The purified proteins of GST-REIN7 and GST-REIN7m expressed in *E*. *coli* were analyzed by an anti-GST antibody.
(TIF)

S9 Fig The conversion of IPA to IAA by YUC8/REIN7 was identified by HPLC profile. (A) The HPLC profile for authentic TAM, IPA and IAA with UV detection (254 nm). (B) The HPLC chromatogram for IAA that was produced from authentic IPA in GST, GST-AtYUC2, GST-REIN7 and GST-REIN7m reaction mixture. (C) The HPLC chromatogram for TAM that was remained in GST, GST-AtYUC2, GST-REIN7 and GST-REIN7m reaction mixture.
(TIF)

S10 Fig The response of Kitaake to different concentrations of auxin. (A) Root phenotypes of the wild type Kitaake treated with various concentrations of IAA. The germinated seed was transferred to MS medium containing various concentrations of IAA and grown in the dark for 3 d. (B) Root length in (A). Each column is the average of 20–30 seedlings and bars indicate ± SD. ** indicates a significant difference compared to 0 μM IAA at *P* < 0.01.
(TIF)

S11 Fig IAA partially rescues the response of root elongation of *oseil1* to ethylene. (A) Partial recovery of the ethylene response of *oseil1* root by IAA. The wild-type and *oseil1* seedlings were grown in the dark for 3 d in the absence or presence of 10 ppm ethylene, with or without supplementation of 10 nM IAA. Bar = 10 mm. (B) Quantification of root inhibition in (A). Each column is the average of 20–30 seedlings. The data are shown as the mean ± SD of three biological replicates. * indicates significant differences between the compared two samples at *P* < 0.05.
(TIF)

S1 Table Primers used in this paper. (XLS)
