# Supplementary figures and images for: The activation of OsEIL1 on YUC8 transcription and auxin biosynthesis is required for ethylene-inhibited root elongation in rice early seedling development
Source: PLoS Genet. 2017 Aug 22;13(8):e1006955. doi: 10.1371/journal.pgen.1006955 (PMC5581195; doi:10.1371/journal.pgen.1006955)

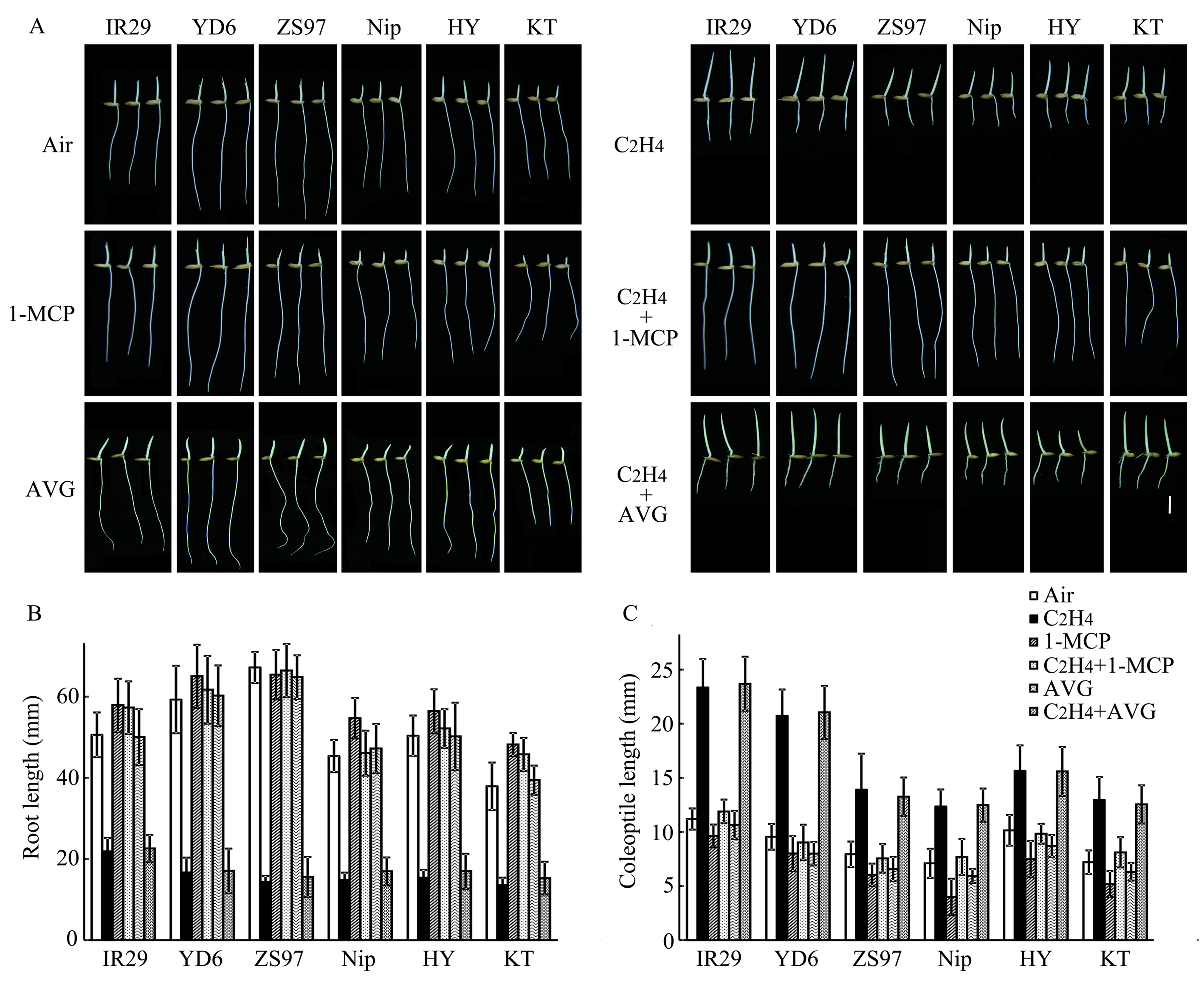

Supplement: S1 Fig — (A) Root and coleoptile phenotypes of japonica (Nip, HY, KT) and indica (IR29, YD6-Yangdao #6, ZS97-Zhenshan 97) cultivars treated with air, 10 ppm ethylene, 1 ppm 1-MCP, 1 ppm 1-MCP plus 10 ppm ethylene, 0.2 μM AVG, and 0.2 μM AVG plus 10 ppm ethylene. Rice seedlings were grown in the dark for 3 d in the presence of various reagents. Bar = 10 mm. (B) Root length for the plants shown in (A). (C) Coleoptile length for the plants shown in (A). Values are shown as the mean ± SD of 20–30 seedlings per genotype. The experiment was repeated at least three times with similar results. (TIF) [file pgen.1006955.s001.tif]

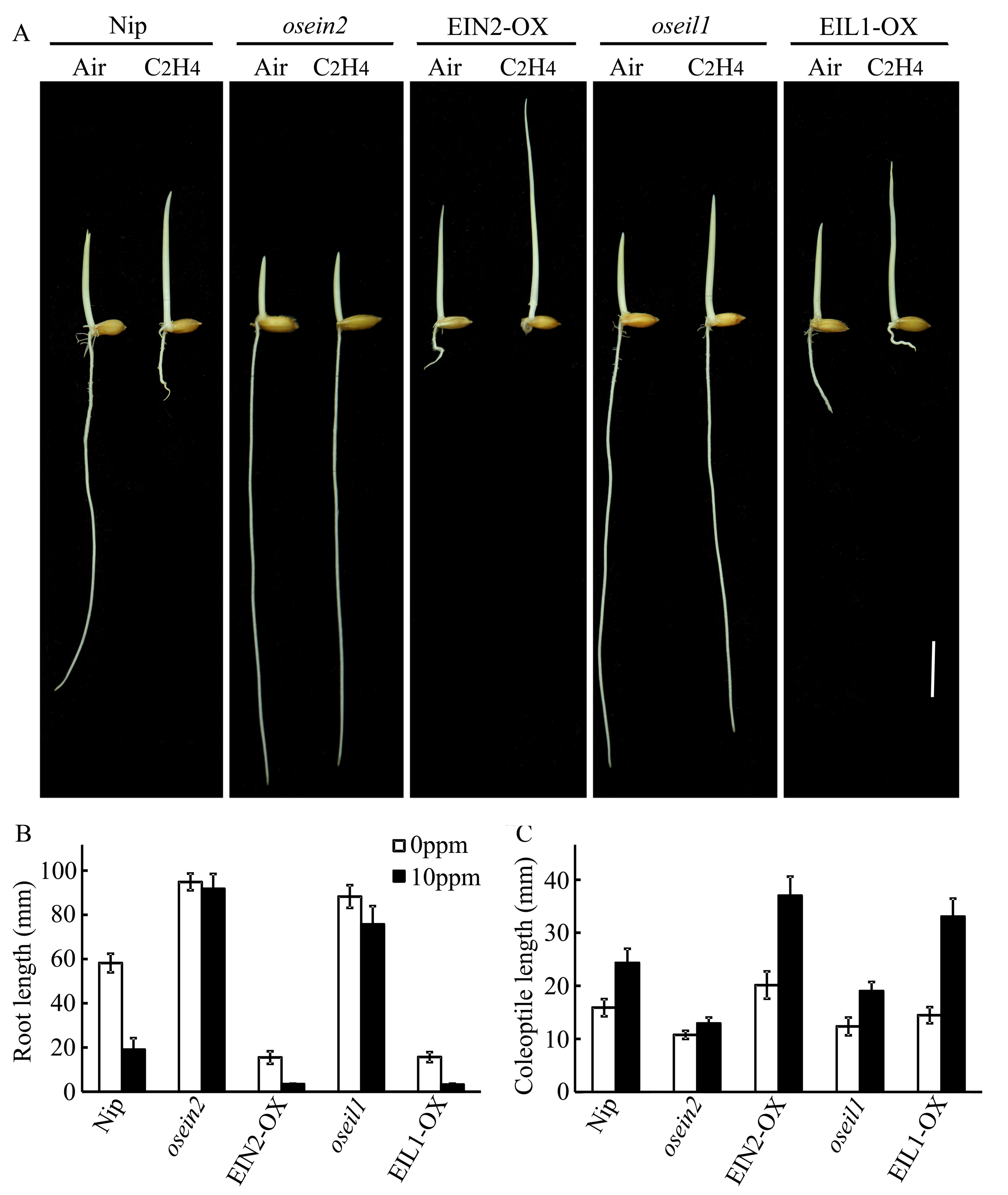

Supplement: S2 Fig — (A) Ethylene-response phenotypes of Nip, osein2, EIN2-OX, oseil1 and EIL1-OX seedlings. The etiolated seedlings were grown in air or 10 ppm ethylene for 3 d. Bar = 10 mm. (B) Root length for the plants shown in (A). (C) Coleoptile length for the plants shown in (A). Values are shown as the mean ± SD of 20–30 seedlings per genotype. The experiment was repeated at least three times with similar results. (TIF) [file pgen.1006955.s002.tif]

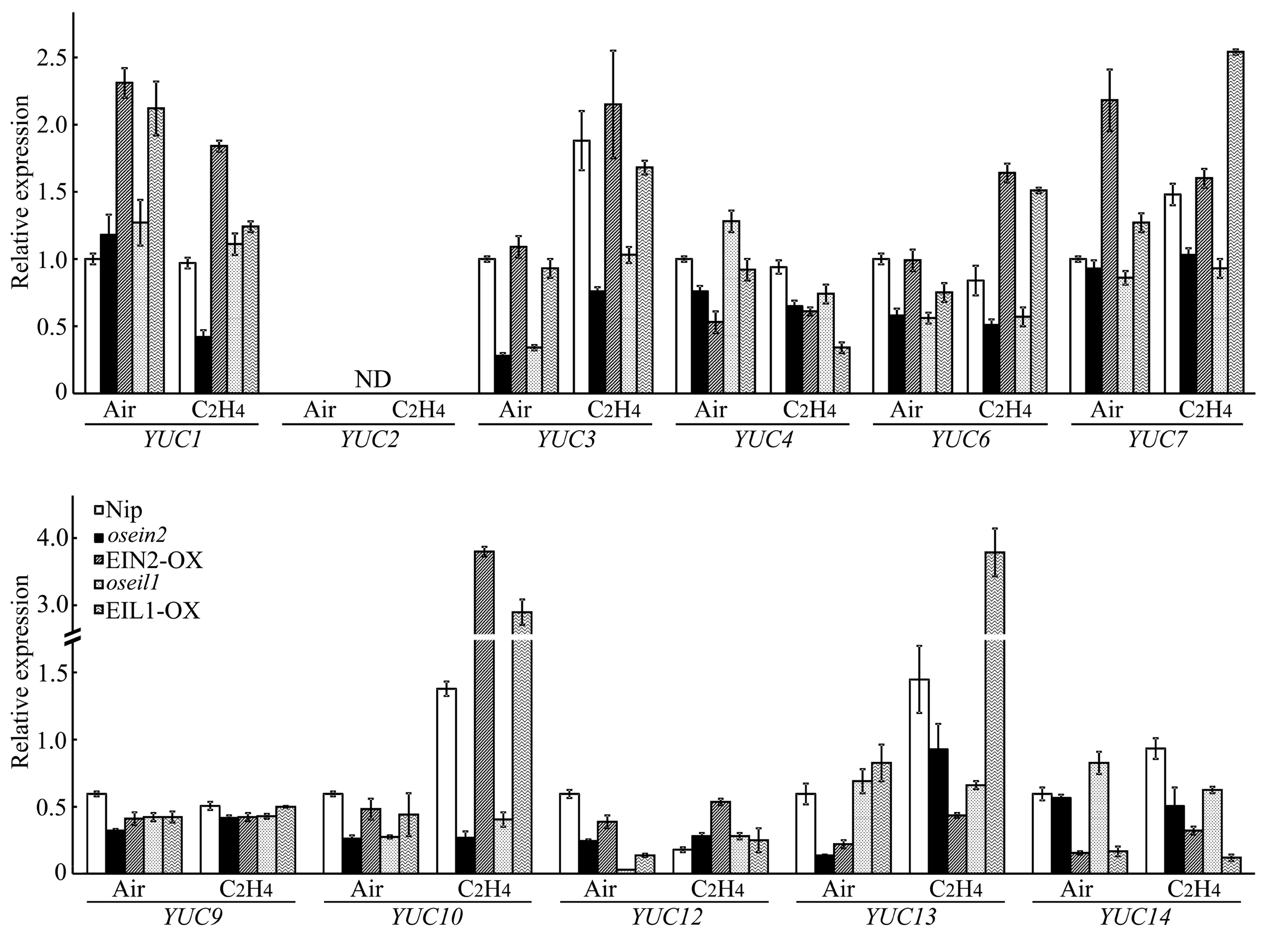

Supplement: S3 Fig — Nip, osein2, EIN2-OX, oseil1 and EIL1-OX seedlings grown in the dark for 3 d and then treated with or without 10 ppm ethylene for 3 h. The RNAs from roots were isolated and used for qPCR. The experiment was repeated at least five times with similar results. ‘ND’ represents not detected. Bars indicate ± SD. (TIF) [file pgen.1006955.s003.tif]

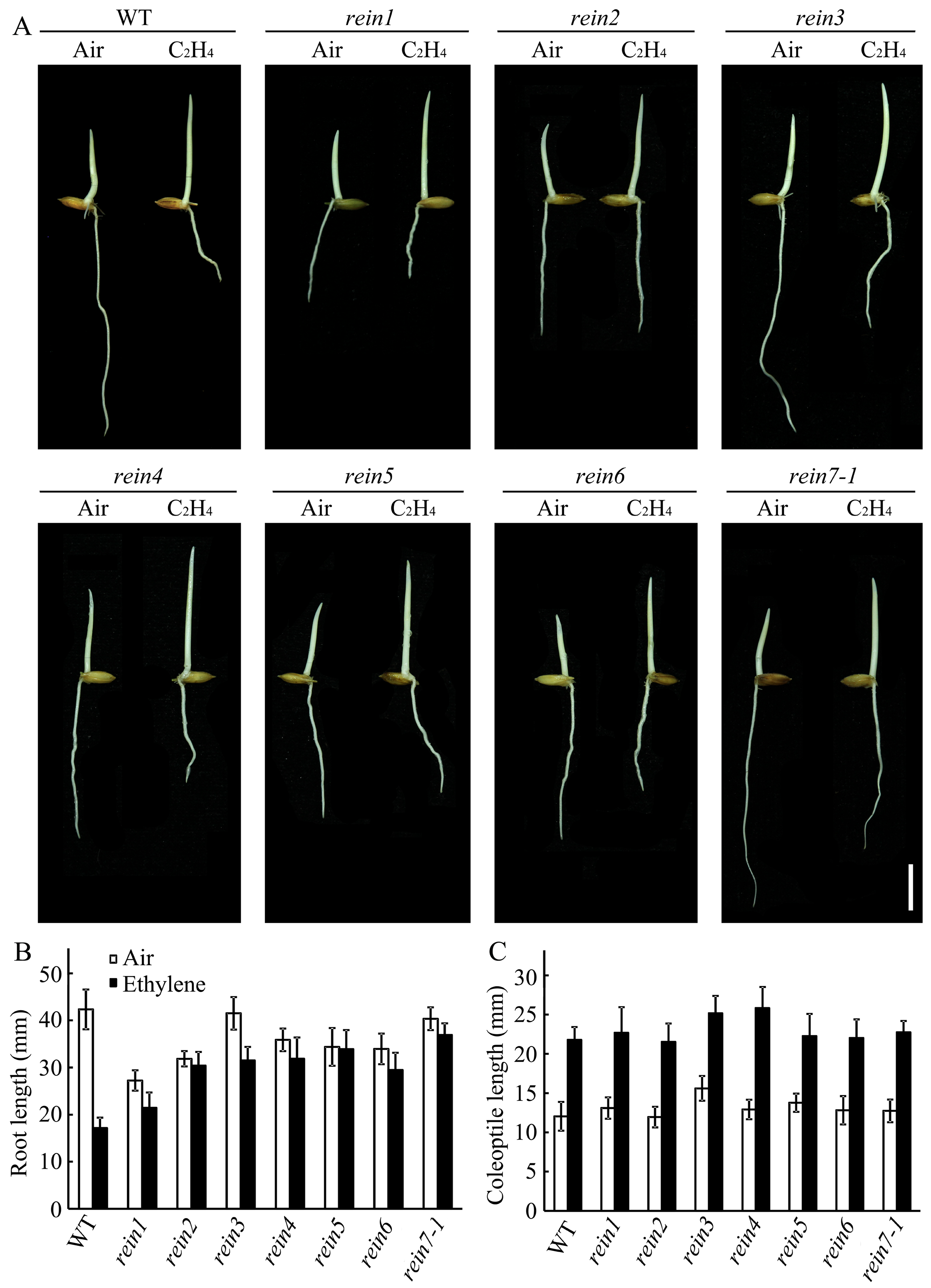

Supplement: S4 Fig — (A) Ethylene-response phenotypes of various rein mutants. The etiolated seedlings were grown in air or 10 ppm ethylene for 3 d. Bar = 10 mm. (B) Root length of the wild type and rein mutants in response to ethylene. (C) Coleoptile length of the wild type and rein mutants in response to ethylene. Each column is the average of 20–30 seedlings, and bars indicate ± SD. (TIF) [file pgen.1006955.s004.tif]

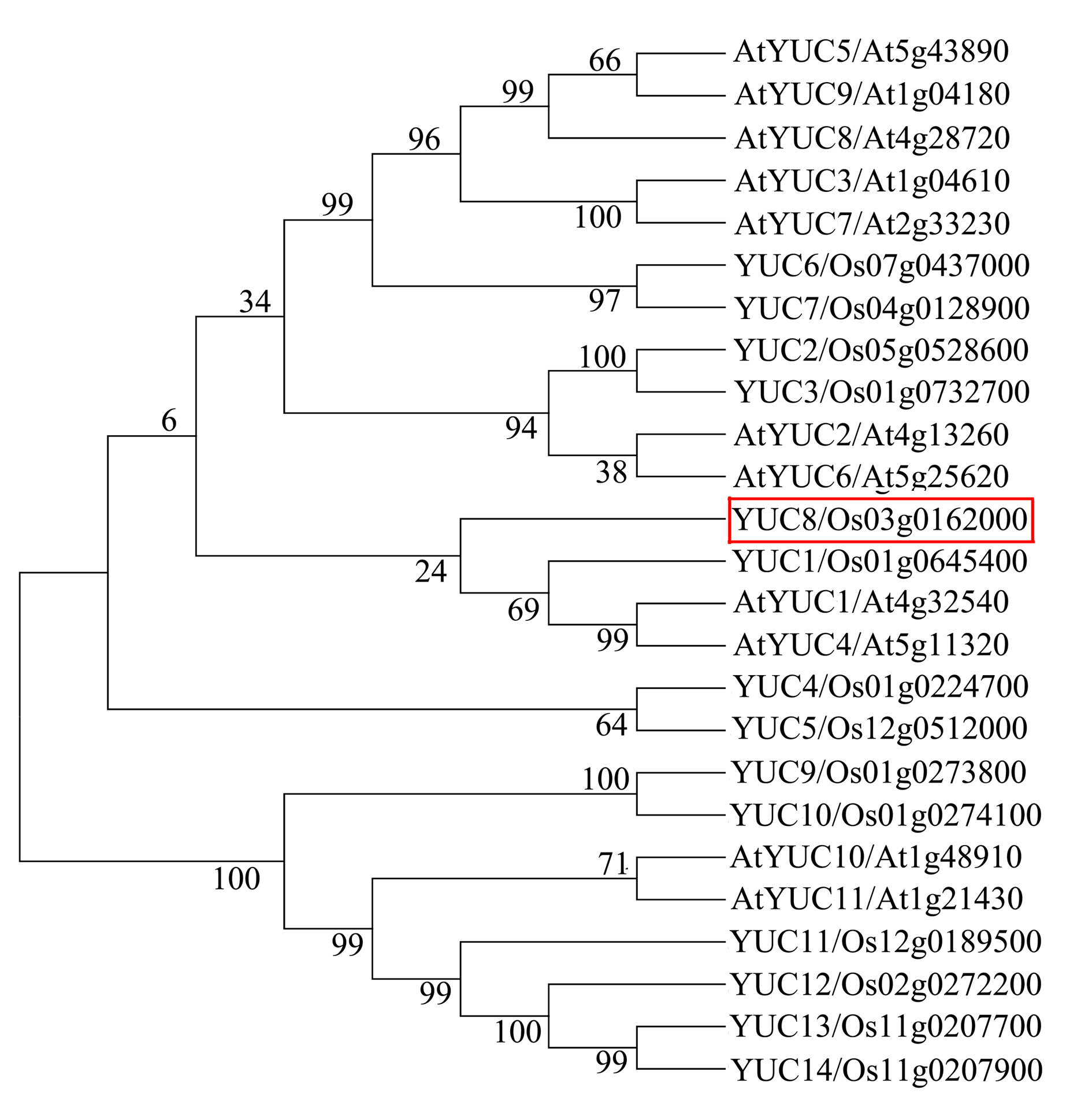

Supplement: S5 Fig — The phylogenetic tree of 14 rice and 11 Arabidopsis YUC genes was constructed using DNAMAN. Bootstrap analysis values are shown at the nodal branches. (TIF) [file pgen.1006955.s005.tif]

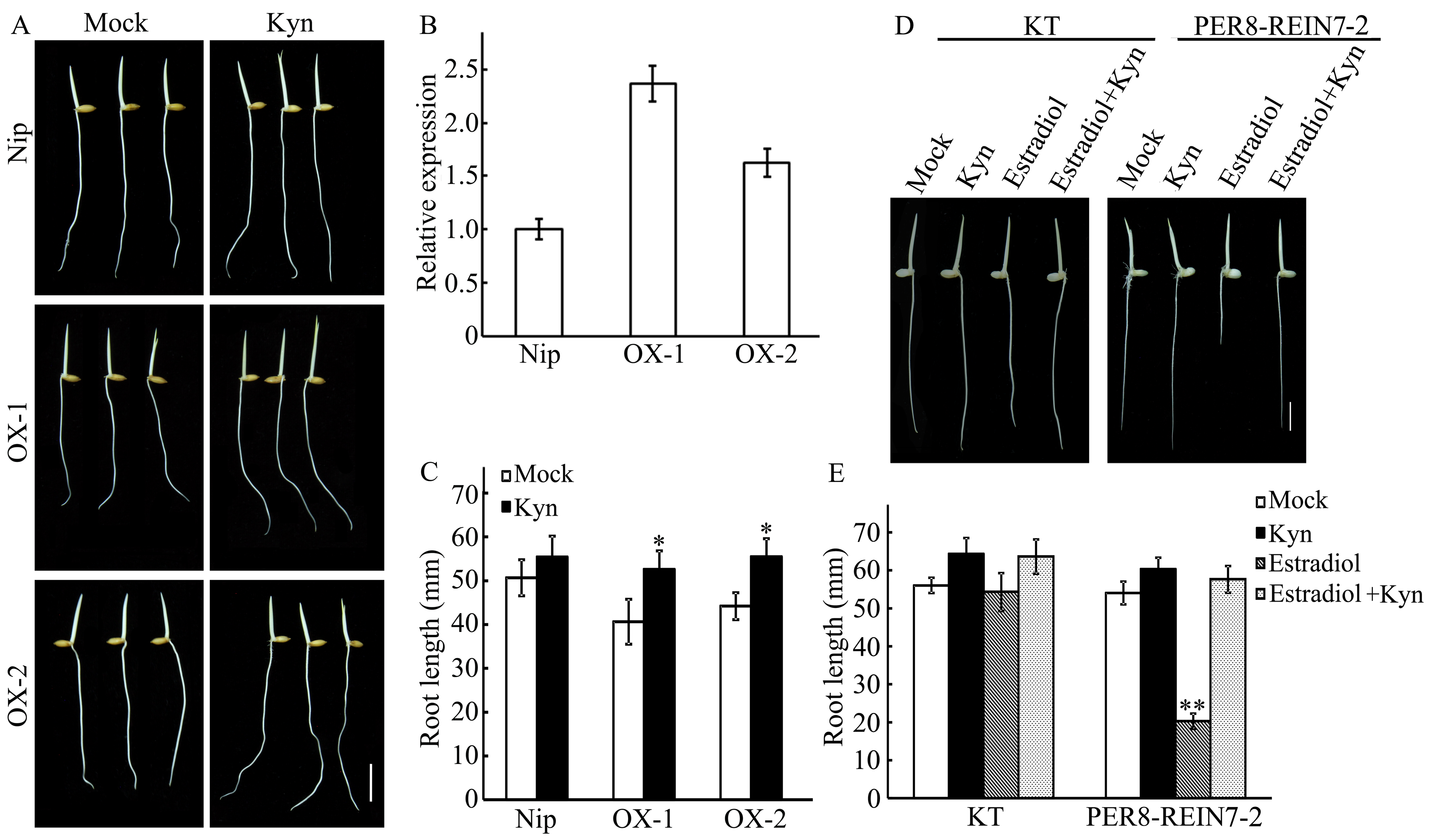

Supplement: S6 Fig — (A) Root phenotype of Nip, constitutive overexpressing YUC8 transgenic (OX-1 and OX-2) etiolated seedlings. The seedlings were grown in the dark for 3 d in the presence or absence of 10 μM Kyn. Bar = 10 mm. (B) YUC8/REIN7 expression in 3-d-old etiolated seedlings. The experiment was repeated at least five times with similar results. Bars indicate ± SD. (C) Root length in (A). (D) Root phenotype of KT and inducible transgenic (PER8-REIN7) etiolated seedlings. The seedlings were grown in the dark for 3 d in the presence or absence of 2.5 μM estradiol, with or without supplementation of 10 μM Kyn. Bar = 10 mm. (E) Root length in (D). In C and E, each column is the average of 20–30 seedlings, and bars indicate ± SD. * and ** indicates a significant difference compared to mock at P < 0.05 and P < 0.01. (TIF) [file pgen.1006955.s006.tif]

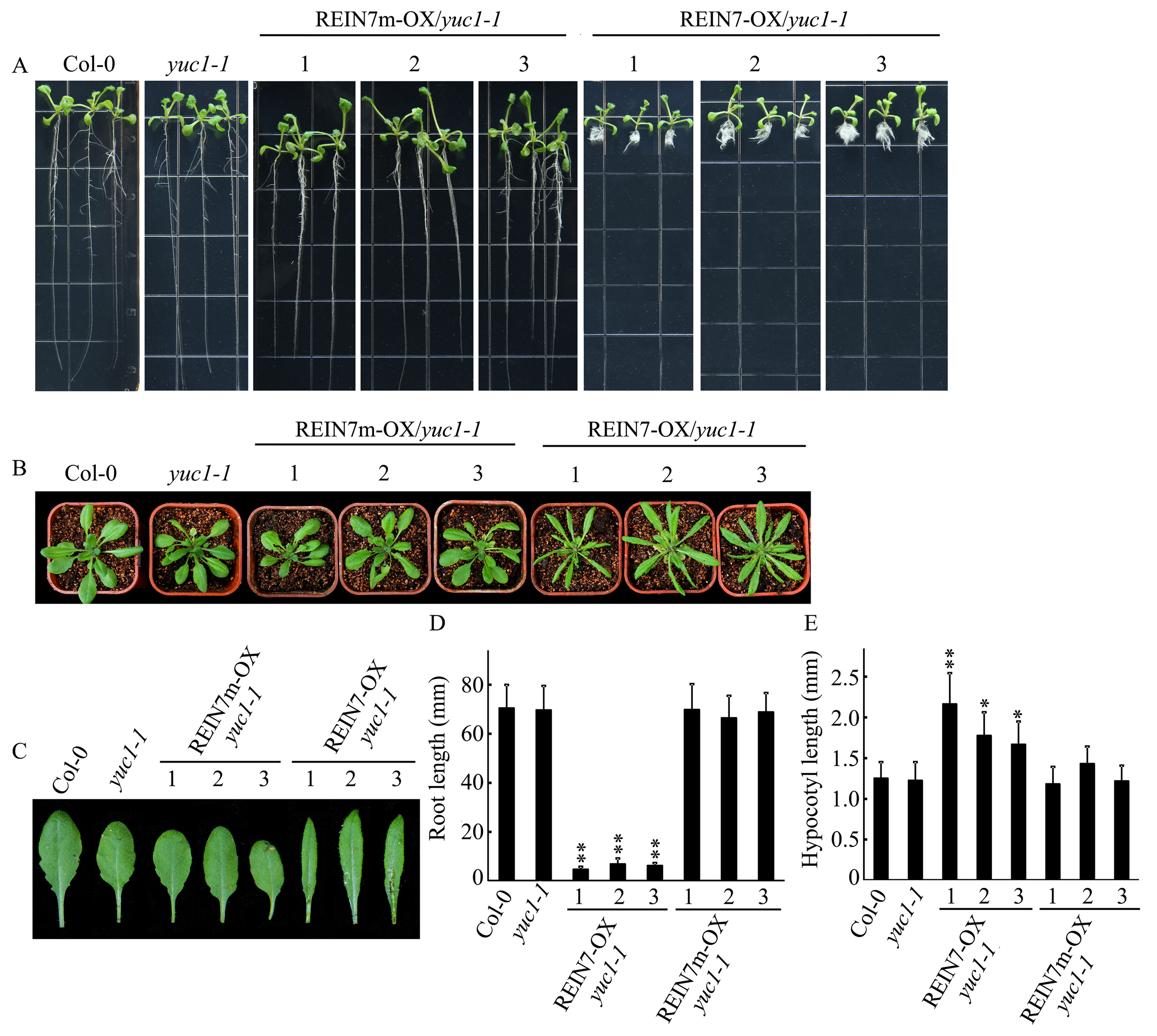

Supplement: S7 Fig — (A) The seedling phenotypes of Col-0, yuc1-1, transgenic lines overexpressing truncated (REIN7m-OX/yuc1-1) or full-length YUC8/REIN7 (REIN7-OX/yuc1-1) in Arabidopsis yuc1-1 mutant grown on MS medium for 7 d. (B) The phenotypes of adult Col-0, yuc1-1, REIN7m-OX/yuc1-1 and REIN7-OX/yuc1-1 lines. (C) Mature leaves of the Col-0, yuc1-1, REIN7m-OX/yuc1-1 and REIN7-OX/yuc1-1 lines. (D) Root length in (A). (E) Hypocotyl length in (A). Each column is the average of 20–30 seedlings and bars indicate ± SD. * and ** indicate significant differences compared to yuc1-1 at P <0.05 and P < 0.01, respectively. (TIF) [file pgen.1006955.s007.tif]

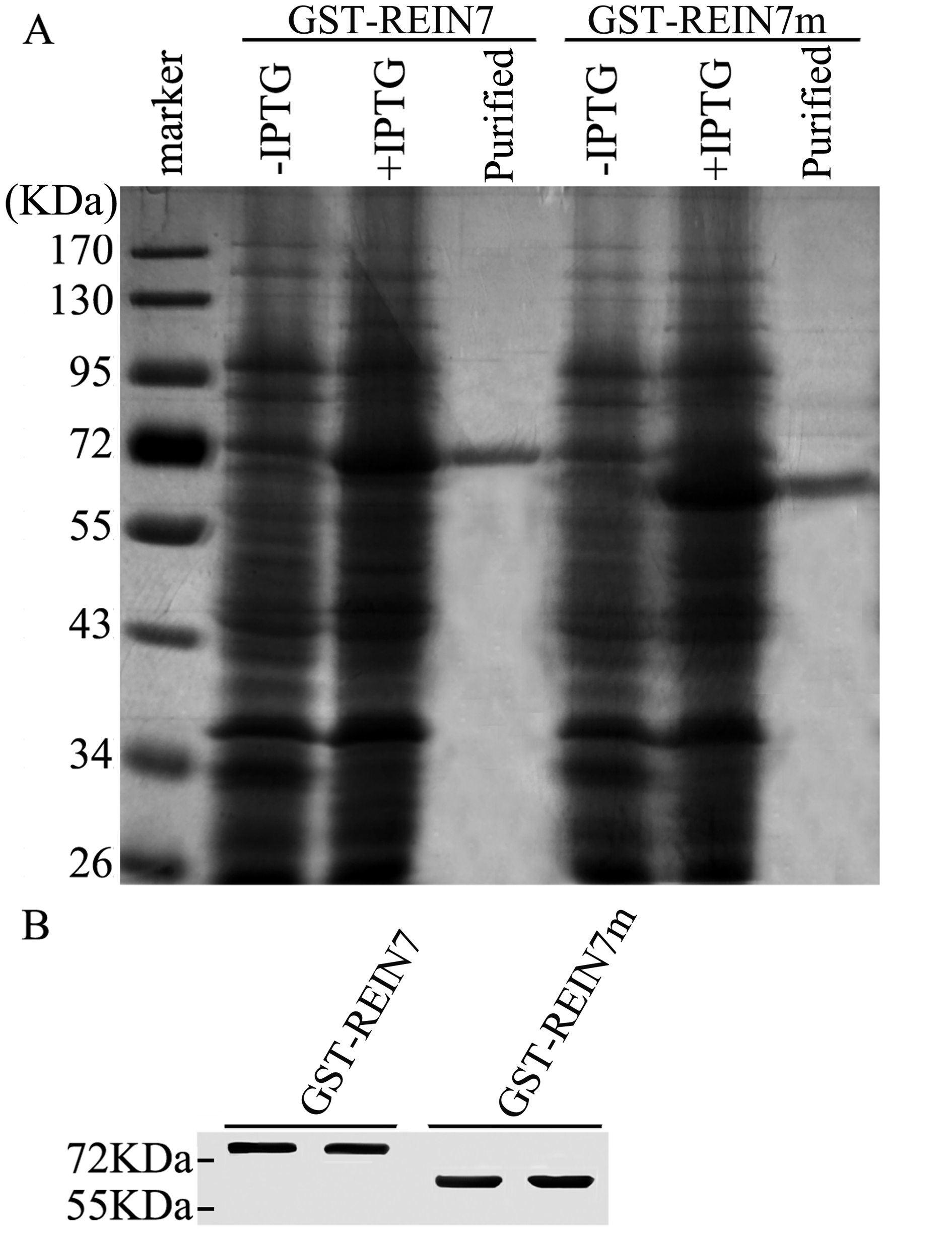

Supplement: S8 Fig — (A) The purified proteins of GST-REIN7 and GST-REIN7m expressed in E. coli were identified by SDS-PAGE electrophoresis.–IPTG and +IPTG: the total proteins from E. coli that were induced or not by IPTG, respectively; Purified: the purified recombined protein. (B) The purified proteins of GST-REIN7 and GST-REIN7m expressed in E. coli were analyzed by an anti-GST antibody. (TIF) [file pgen.1006955.s008.tif]

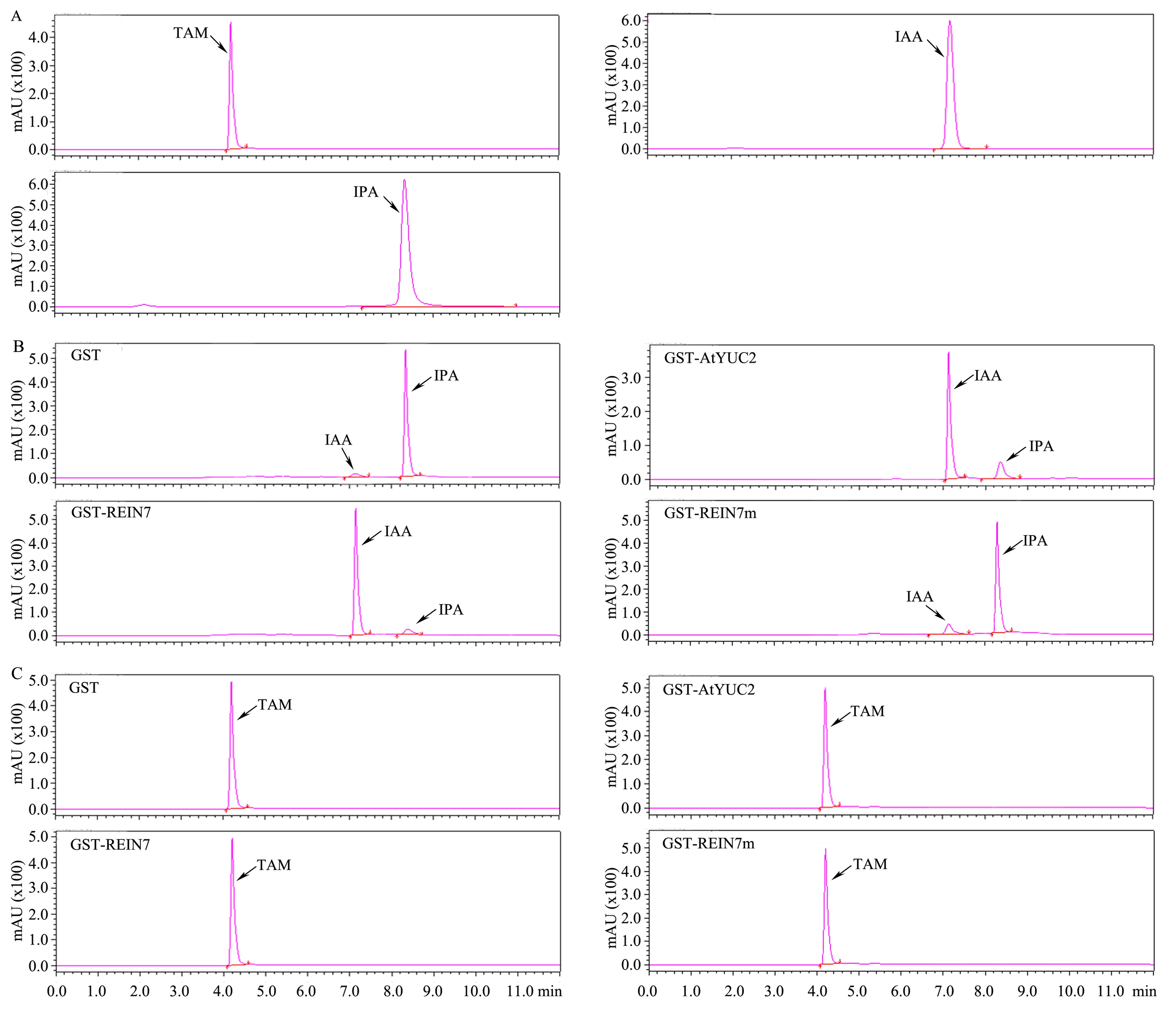

Supplement: S9 Fig — (A) The HPLC profile for authentic TAM, IPA and IAA with UV detection (254 nm). (B) The HPLC chromatogram for IAA that was produced from authentic IPA in GST, GST-AtYUC2, GST-REIN7 and GST-REIN7m reaction mixture. (C) The HPLC chromatogram for TAM that was remained in GST, GST-AtYUC2, GST-REIN7 and GST-REIN7m reaction mixture. (TIF) [file pgen.1006955.s009.tif]

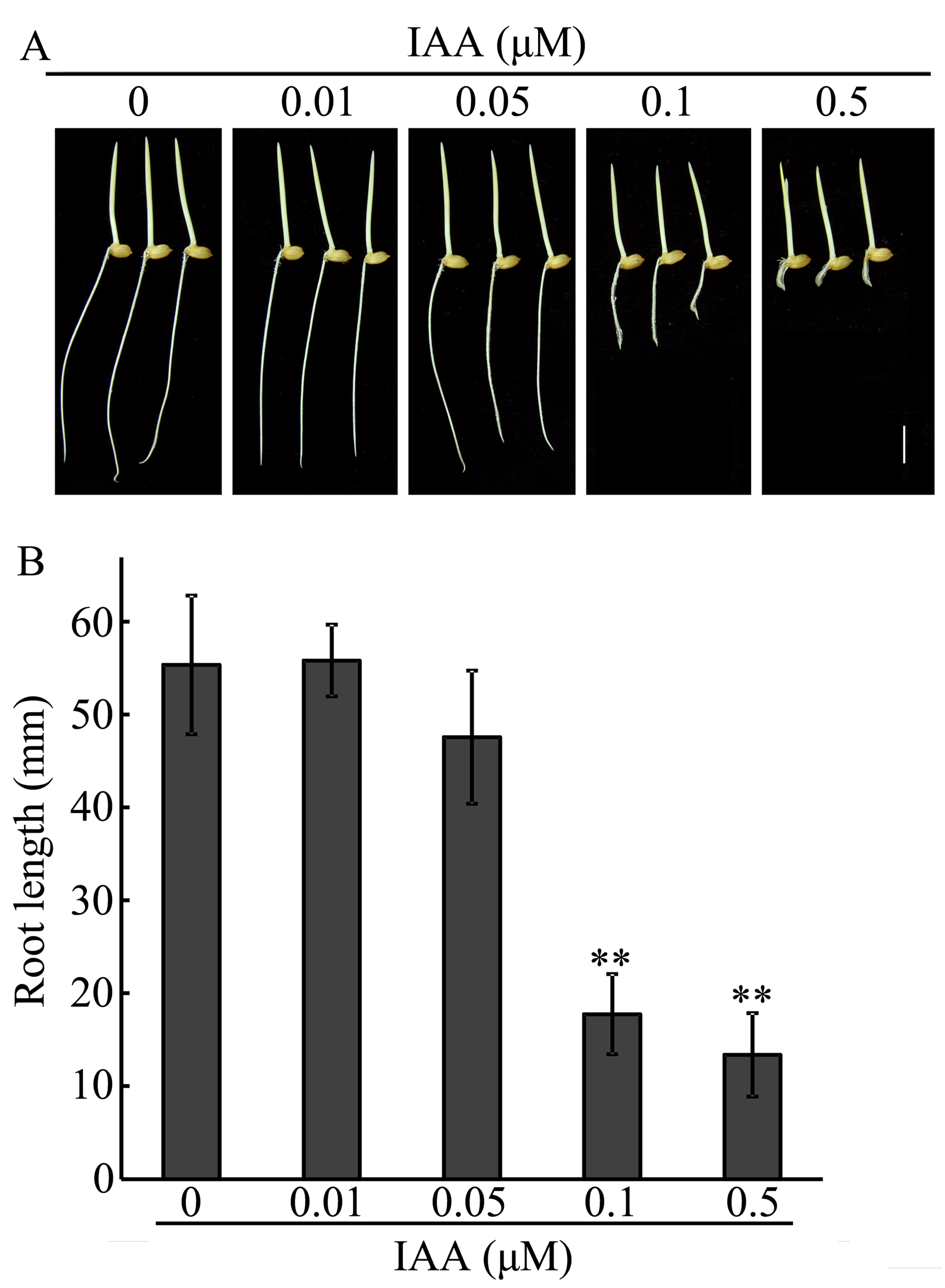

Supplement: S10 Fig — (A) Root phenotypes of the wild type Kitaake treated with various concentrations of IAA. The germinated seed was transferred to MS medium containing various concentrations of IAA and grown in the dark for 3 d. (B) Root length in (A). Each column is the average of 20–30 seedlings and bars indicate ± SD. ** indicates a significant difference compared to 0 μM IAA at P < 0.01. (TIF) [file pgen.1006955.s010.tif]

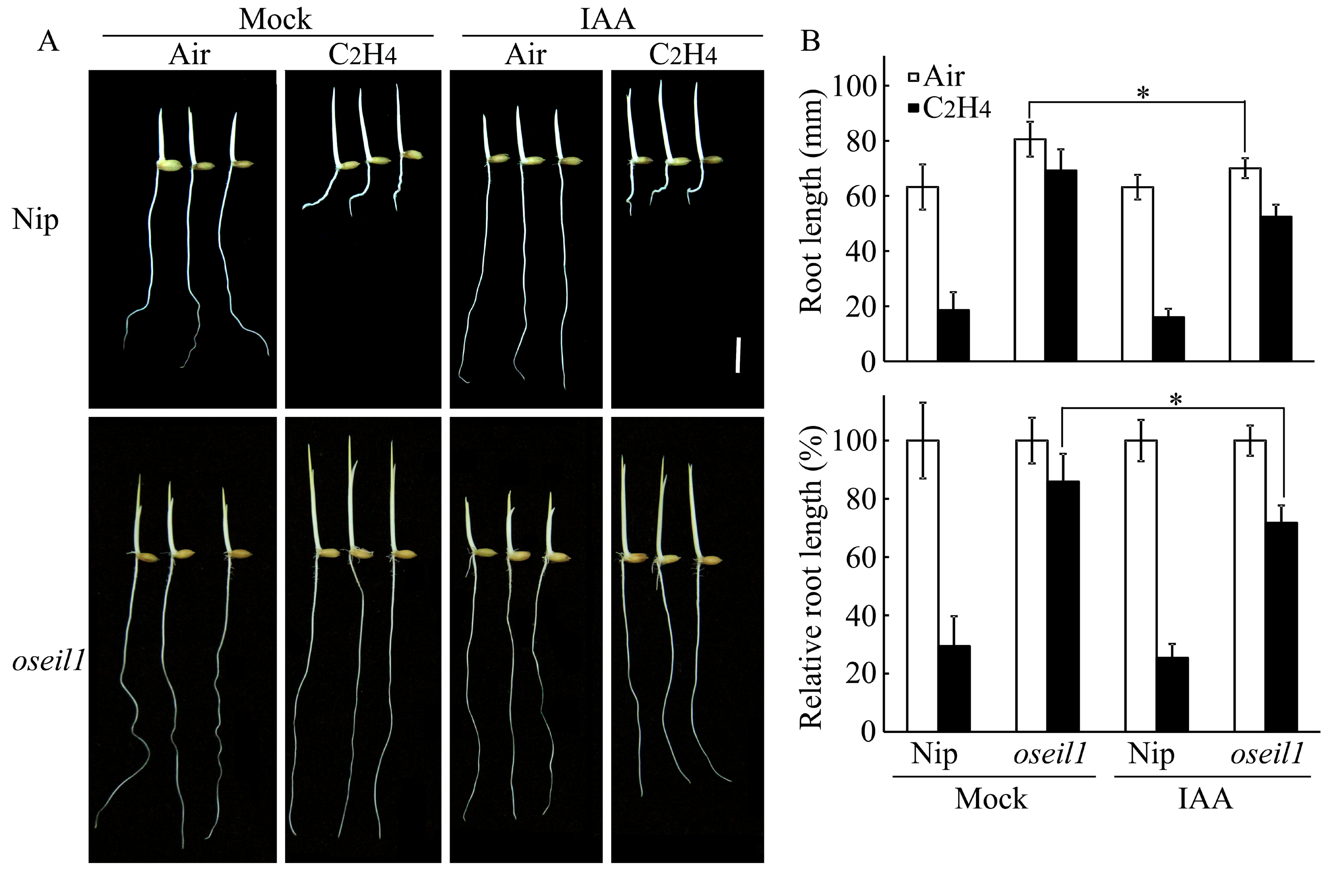

Supplement: S11 Fig — (A) Partial recovery of the ethylene response of oseil1 root by IAA. The wild-type and oseil1 seedlings were grown in the dark for 3 d in the absence or presence of 10 ppm ethylene, with or without supplementation of 10 nM IAA. Bar = 10 mm. (B) Quantification of root inhibition in (A). Each column is the average of 20–30 seedlings. The data are shown as the mean ± SD of three biological replicates. * indicates significant differences between the compared two samples at P < 0.05. (TIF) [file pgen.1006955.s011.tif]
